# Particular genomic and virulence traits associated with preterm infant-derived toxigenic *Clostridium perfringens* strains

**DOI:** 10.1038/s41564-023-01385-z

**Published:** 2023-05-25

**Authors:** Raymond Kiu, Alexander G. Shaw, Kathleen Sim, Antia Acuna-Gonzalez, Christopher A. Price, Harley Bedwell, Sally A. Dreger, Wesley J. Fowler, Emma Cornwell, Derek Pickard, Gusztav Belteki, Jennifer Malsom, Sarah Phillips, Gregory R. Young, Zoe Schofield, Cristina Alcon-Giner, Janet E. Berrington, Christopher J. Stewart, Gordon Dougan, Paul Clarke, Gillian Douce, Stephen D. Robinson, J. Simon Kroll, Lindsay J. Hall

**Affiliations:** 1 Gut Microbes and Health, Quadram Institute Bioscience, Norwich, UK; 2 Faculty of Medicine, Imperial College London, London, UK; 3 Department of Medicine, University of Cambridge, Cambridge, UK; 4 Neonatal Intensive Care Unit, The Rosie Hospital, Cambridge, UK; 5 Hub for Biotechnology in the Built Environment, Northumbria University, Newcastle upon Tyne, UK; 6 Translational and Clinical Research Institute, Newcastle University, Newcastle upon Tyne, UK; 7 Newcastle Neonatal Services, Newcastle upon Tyne NHS Foundation Trust, Newcastle upon Tyne, UK; 8 Norfolk and Norwich University Hospital, Norwich, UK; 9 Norwich Medical School, University of East Anglia, Norwich, UK; 10 Institute of Infection, Immunity and Inflammation, College of Medical, Veterinary and Life Sciences, University of Glasgow, Glasgow, UK; 11 School of Biological Sciences, University of East Anglia, Norwich, UK; 12 Intestinal Microbiome, School of Life Sciences, ZIEL—Institute for Food & Health, Technical University of Munich, Freising, Germany

**Keywords:** Microbiology, Molecular biology

## Abstract

*Clostridium perfringens* is an anaerobic toxin-producing bacterium associated with intestinal diseases, particularly in neonatal humans and animals. Infant gut microbiome studies have recently indicated a link between *C. perfringens* and the preterm infant disease necrotizing enterocolitis (NEC), with specific NEC cases associated with overabundant *C. perfringens* termed *C. perfringens*-associated NEC (CPA-NEC). In the present study, we carried out whole-genome sequencing of 272 *C. perfringens* isolates from 70 infants across 5 hospitals in the United Kingdom. In this retrospective analysis, we performed in-depth genomic analyses (virulence profiling, strain tracking and plasmid analysis) and experimentally characterized pathogenic traits of 31 strains, including 4 from CPA-NEC patients. We found that the gene encoding toxin perfringolysin O, *pfoA*, was largely deficient in a human-derived hypovirulent lineage, as well as certain colonization factors, in contrast to typical *pfoA*-encoding virulent lineages. We determined that infant-associated *pfoA*^+^ strains caused significantly more cellular damage than *pfoA*^−^ strains in vitro, and further confirmed this virulence trait in vivo using an oral-challenge C57BL/6 murine model. These findings suggest both the importance of *pfoA*^+^ *C. perfringens* as a gut pathogen in preterm infants and areas for further investigation, including potential intervention and therapeutic strategies.

## Abstract (web summary)

Genomic and phenotypic analyses of 272 infant-associated *Clostridium perfringens* isolates suggest that *pfoA* is an important toxin gene in *C. perfringens* linked with preterm infant intestinal diseases.

## Main

*C. perfringens* has been frequently linked to various intestinal diseases in both humans and animals, particularly in neonates^1^. It is known to secrete >20 toxins, including several pore-forming toxins, for example, β-toxin, perfringolysin O (PFO), NetB and *C. perfringens* enterotoxin (CPE), alongside encoding other virulence factors associated with different facets of pathophysiology^2–6^. Although whole-genome sequencing (WGS) has advanced our understanding of adult-associated *C. perfringens*, gastroenteritis-associated strains, association studies with other intestinal diseases in younger age groups, including carriage in healthy individuals, are very limited^7–9^.

NEC, the most severe and lethal neonatal gastrointestinal (GI) emergency worldwide, is an acquired inflammatory gut disease that manifests as tissue necrosis in the GI tract^10,11^. NEC typically affects 5–15% of very-low-birthweight (<1,500 g) preterm infants, leading to high mortality rates (~40%) and severe longer-term complications^12–15^. Clinical observations support a microbial role in NEC, including gas cyst formation in the gut wall (pneumatosis intestinalis), and antimicrobial treatment often resolves symptoms^16–18^. Several pathogens including *Klebsiella* spp. and *C. perfringens* have been linked to clinical NEC^19,20^, with microbiota-based, preterm infant cohort studies implicating overabundance before NEC^21,22^. *C. perfringens* has been consistently reported as a potential pathogenic agent associated with NEC^1,23,24^, with CPA-NEC symptoms often more fulminant and severe compared with ‘classic’ NEC^19,25,26^.

In the present study, we describe an extensive genomic study of *C. perfringens* clinical preterm isolates from non-CPA-NEC (95%) and CPA-NEC patients (5%). We probed virulence factors and plasmids associated with infant/CPA-NEC *C. perfringens* strains and traced putative hospital transmission. Additional experiments on a selection of *C. perfringens* strains and an oral-challenge infection model allowed us to assay key virulence and pathology readouts.

## Results

### Eight distinct phylogenetic lineages of *C. perfringens*

**Fig. 1:**
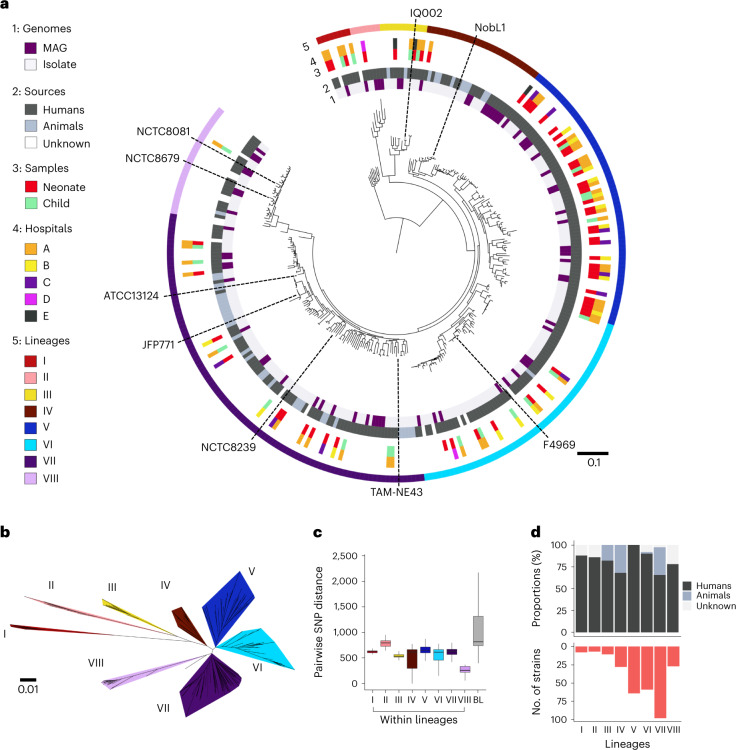
Strain-level population genomics of *C. perfringens* reveals eight distinct phylogenetic lineages. **a**, Mid-point rooted, maximum-likelihood phylogenetic tree of 302 representative *C. perfringens* strains (derived from a collection of 673 *C. perfringens* genomes) inferred from 8,506 SNPs (from 858 single-copy core genes), aligned with metadata including genome types, sources, sample hosts, hospitals and lineages assigned using hierBAPS. Black dashed lines, type strain ATCC13124 and several representative strains were labelled in this phylogenetic tree. Branch lengths are indicative of the estimated nucleotide substitution per site (SNPs). MAG, metagenome-assembled genomes (from MGnify study, human gut microbiome-derived *C. perfringens* genomes only). **b**, Unrooted phylogenetic tree coloured by assigned lineages. **c**, Pairwise SNP distances among strains located within lineages I (*n* = 8), II (*n* = 7), III (*n* = 11), IV (*n* = 28), V (*n* = 57), VI (*n* = 66), VII (*n* = 98) and VIII (*n* = 27) and between lineages. The box in the boxplot represents 50% of the central data, in between the lower and upper quartiles, with a central line representing the median, whereas the whiskers show the most extreme data points. BL, between lineages. **d**, Proportions of human and animal-derived *C. perfringens* genomes in each lineage. Source data

We isolated and performed WGS on 272 *C. perfringens* isolates from infant stool samples collected longitudinally from 70 individuals (including 4 CPA-NEC infants), obtained from 5 UK neonatal intensive care units (NICUs) (and at-home collections) between 2011 and 2016 (Supplementary Table 1), which indicated an ~24% incidence. A strain-level, core-genome, global phylogenetic tree (isolates and metagenome-assembled genomes; Fig. 1a) clustered into eight major lineages (Fig. 1b,c). The relatively distant lineage VIII consisted of mostly typical human gastroenteritis isolates, whereas lineage VII included mixed human and animal sources, indicating a potential zoonotic signature. Lineage V comprised exclusively human-derived strains (Fig. 1d), with more than half obtained from infants and hospital linked.

### Genomic investigation reveals a hypovirulent lineage

**Fig. 2:**
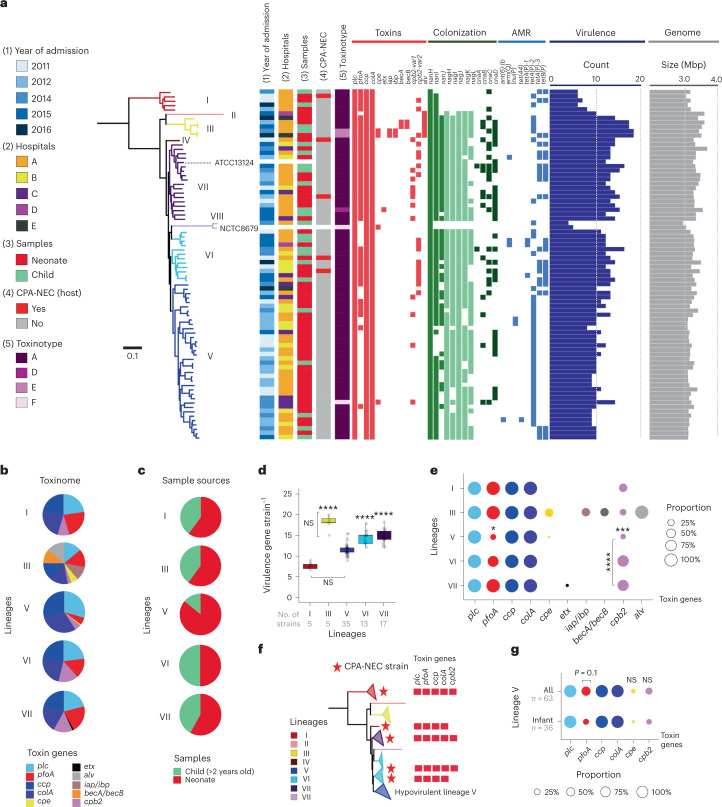
Genomic analysis of infant-associated *C. perfringens* virulence potentials unravels a hypovirulent lineage that does not commonly encode toxin genes *pfoA* and *cpb2*. **a**, A mid-point-rooted, maximum-likelihood phylogenetic tree of 80 infant-associated *C. perfringens* representative strains, derived from 272 *C. perfringens* isolate genomes using an ANI cutoff of 99.9% (strain-level) for dereplication of genomes. This tree was inferred from 175,479 core SNPs (in 1,995 single-copy core genes) aligned with clinical metadata and virulence profiles of each strain including toxin genes, colonization factors and antimicrobial resistance genes, in addition to virulence gene counts and genome sizes, as indicated. Branches are colour coded according to the lineages assigned in Fig. 1. AMR, antimicrobial resistance. **b**, Toxinome profiles of each lineage. **c**, Sample source proportions in each lineage. **d**, Virulence count (per strain) comparison across lineages. Significance was pairwise compared. The box in the boxplot represents 50% of the central data, in between the lower and upper quartiles, with a central line representing the median, whereas the whiskers show the most extreme data points. There was no difference between lineages I and V, whereas lineages III, VI and VII (no difference among these three lineages) are significantly different from lineages I and V. ^****^P < 0.0001. Statistical analysis was performed using Kruskal–Wallis test and post-hoc Dunn’s test. **e**, Proportion of each toxin gene across all lineages. The significance was pairwise compared within individual toxin genes. The significant difference was indicated in *cpb2* genes across lineages V, VI and VII, and pairwise comparison indicated that lineage V has reduced proportionally in *cpb2* genes compared with lineages VI and VII. ^***^P = 0.003, ^****^P < 0.0001, whereas proportion of *pfoA* gene is significantly reduced in lineage V versus all other lineages: ^*^P < 0.05. **f**, Full toxin gene profiles of five CPA-NEC *C. perfringens* strains. **g**, Toxin gene proportion in isolates within global lineage V (which includes MAGs from human microbiome project) and infant-associated lineage V isolates. NS, non-significant (Note: lineages II, IV and VIII were not included in statistical investigations due to these lineages only encompassing singletons.) In **e** and **g**, statistical analysis was performed using Fisher’s test (two sided). Source data

**Extended Data Fig. 1:**
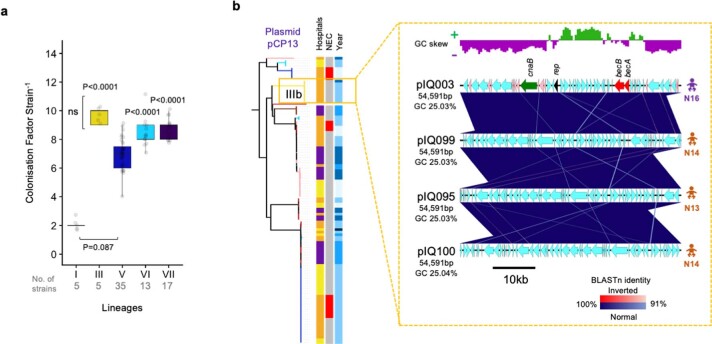
Genomic analysis of *C. perfringens* strains. **a**, Colonisation factor comparison across all isolates by lineage. Colonisation factors are significantly more in lineages III, VI and VII than lineages I and V, while no significant difference between lineages III, VI and VII, similarly also lineages I and V. The box in boxplot represents 50% of the central data, in between lower and upper quartile, with a central line representing the median while whiskers show the most extreme data points. Statistical analyses were performed with Kruskal-Wallis test and post-hoc Dunn’s test of multiple comparison with P values adjusted by Benjamini-Hochberg method. **b**, Comparative genomic architecture analysis of identical plasmids from sub-lineage IIIb isolates. Source data

Genome-based, strain-level virulence profiling was performed on all 80 infant-associated *C. perfringens* representative strains (derived from original 272, including reference strains; Fig. 2a). Toxinome analysis indicated that lineage III encoded more toxin genes (*n* = 9) than other lineages, whereas lineage V had a reduced presence of the pore-forming toxin gene *pfoA* (Fig. 2b) and mostly consisted of neonatal isolates (Fig. 2c). Overall, *C. perfringens* strains from lineages III, VI and VII carried significantly more virulence genes versus strains from lineages I and V (Fig. 2d). Significantly fewer toxin genes including *pfoA* and *cpb2* were encoded within lineage V versus other lineages (Fig. 2e), with colonization-associated genes sialidases (*nan*), hyaluronidase (*nag*) and adhesin (*cna*) overrepresented in lineages III, VI and VII when compared with lineages I and V (Extended Data Fig. 1a).

CPA-NEC strains (*n* = 5) nested within lineages I, IV, VI and VII, respectively (Fig. 2f), and encoded similar toxinome profiles. No CPA-NEC strains nested within lineage V, which predominantly contained strains with fewer virulence potentials (including apparent lack of *pfoA*), indicative of a potentially hypovirulent or commensal-like lineage (Fig. 2g).

### SNP analysis indicates potential strain dissemination

**Fig. 3:**
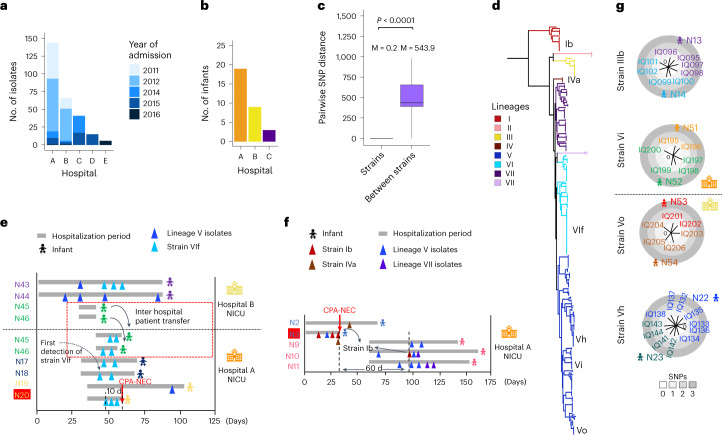
In-depth strain-level SNP analysis in tracking putative transmission of infant-associated *C. perfringens*. **a**, Number of *C. perfringens* isolates sequenced from five hospitals. **b**, Number of infants with two or more longitudinal sampling time points. **c**, Pairwise SNP distance in longitudinally sampled isolates (*n* = 209): strains (ANI > 99.9%) versus between strains (ANI < 99.9%). M, mean (SNPs). The statistical analysis was performed using Wilcoxon’s test (two sided). The box in the boxplot represents 50% of the central data, in between the lower and upper quartiles, with a central line representing the median, whereas the whiskers show the most extreme data points. **d**, Phylogenetics of 272 infant-associated *C. perfringens* isolates with branches colour coded according to the lineage assigned. The key strains, defined as isolates having highly similar ANI > 99.9%, as indicated on the tree, are further described in **e**–**g**. **e**, Tracking multi-host *C. perfringens* strain VIf (31 isolates) which involves six individuals in two sister hospitals, A and B, based on clinical metadata. Twin pairs are colour coded. A CPA-NEC infant (N20) was highlighted in red. **f**, Putative intrahospital circulation of strain Ib. Strain Ib was not detected in a 60-d window in the infant cohort. A CPA-NEC infant (N3) was highlighted in red. **g**, Comparison of SNP distance trees between isolates from twin pairs, suggesting dissemination of *C. perfringens*. Source data

We investigated circulation of *C. perfringens* strains based on 210 longitudinal isolates (≥2 sampling time points) obtained from 31 infants from 3 hospitals (Fig. 3a,b). Strain-level SNP distance was determined to be ~0.2 SNPs, whereas between-strain SNP distance was estimated at ~544 SNPs (Fig. 3c).

Sublineage VIf represented a clonal cluster consisting of 31 identical isolates at core-SNP level (0 SNPs) obtained from 6 infants residing in sister hospitals A and B (Fig. 3d,e). Clinical metadata linkage indicated that the index case of strain VIf was in infant N18 in hospital A. Later, this strain was detected in several individuals in hospital A (N17, N18 and N20), one of whom (N20) was diagnosed with CPA-NEC. Infant N20, who was also diagnosed with a condition predisposing to GI mucosal hypoxaemia, subsequently succumbed to NEC 10 d after strain VIf was first detected. N17 and N18 (index case for strain VIf) had an episode of possible NEC which led to administration of antibiotics (broad-range β-lactam + vancomycin) for 2 d, with strain VIf first detected ~8 d after antibiotic administration finished in N18. Strain VIf was also detected in individuals residing in hospital B (across the same 100-d window). This suggests potential spread of the *C. perfringens* strain between hospitals, whether transferred on/in patients, their parents or equipment or healthcare staff. We also observed that strain Ib in the index case N3 (who succumbed on day 27; Fig. 3f) was again detected in N10 (after a 60-d gap), suggesting possible horizontal transmission and persistence of *C. perfringens* strains, potentially in the NICU environment. Additional transmissions events were also implicated in four twin pairs carrying identical strains (Fig. 3g).

### Virulence plasmid carriage among infant-associated isolates

**Fig. 4:**
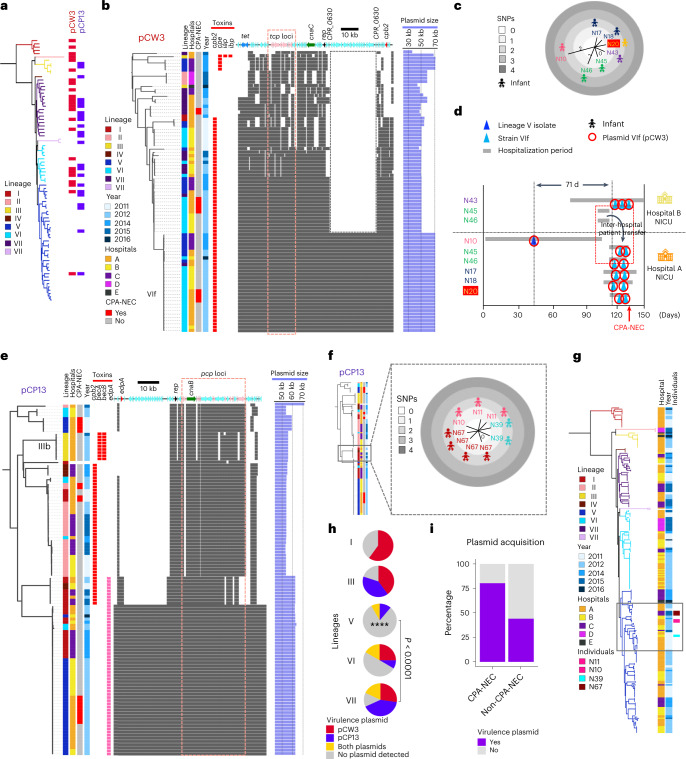
Computational analysis of key conjugative virulence plasmids pCW3 and pCP13 encoded by infant-associated *C. perfringens* isolates. **a**, Carriage/distribution of pCW3 and pCP13 conjugative virulence plasmids among infant-associated *C. perfringens* strains indicated in the infant *C. perfringens* phylogeny. **b**, Plasmid-coding sequences mapped to reference plasmid sequence (pLH112) to present a comparative overview of pCW3 plasmids computationally extracted from infant-associated *C. perfringens* isolates (*n* = 85). Data/sequences were aligned with a newly constructed plasmid phylogeny. **c**, SNP distance tree of plasmids from strain VIf (from seven individuals). **d**, Probable plasmid circulation map of plasmid VIf (identical plasmids encoded in strains VIf). **e**, Plasmid-coding sequences mapped to reference plasmid sequence (pLH112) to present a comparative overview of pCP13 plasmids computationally extracted from infant-associated *C. perfringens* isolates (*n* = 111). Data/sequences were aligned with a newly constructed plasmid phylogeny. **f**, SNP distance tree of identical plasmids encoded in nine isolates obtained from four individuals. **g**, Isolates’ positions from the four individuals indicated in **f** within the infant-associated *C. perfringens* phylogenetic tree. These four individuals harboured *C. perfringens* isolates (*n* = 9; from different hospitals) that encoded identical pCP13 conjugative virulence plasmids nested within distinct sublineages of lineage V. **h**, Proportions of conjugative virulence plasmids encoded by *C. perfringens* strains across lineages. Proportion of strain-level virulence plasmid carriage was significantly lower in lineage V compared with lineage VII (^****^P < 0.0001). Statistical analysis was performed using Fisher’s test (two sided). **i**, Proportions of infant-associated *C. perfringens* strains carrying one or more conjugative virulence plasmid(s), pCW3 and/or pCP13: a comparison across lineage V (*n* = 36), CPA-NEC strains (*n* = 5) and non-CPA-NEC strains (*n* = 73). Statistical analysis was performed using Fisher’s test (two sided). The proportional difference was not found to be significant. Source data

We next investigated plasmid acquisition of two key *C. perfringens* (virulence) conjugative plasmid families, pCW3 (ref. ^27^) and pCP13 (ref. ^28^) (Fig. 4a). Plasmid pCW3 (with *tcp* system) encoded toxin genes such as *cpb2* and *cpe*, iota binary toxin genes *iap* and *ibp* and tetracycline resistance determinants *tetA(P)* and *tetB(P)* (Fig. 4b). The adhesin gene *cnaC* was also detected in most pCW3 plasmids, potentially enhancing colonization capacity. NEC-associated isolates from sublineage VIf primarily carried pCW3 plasmids in either ~50-kb or ~70-kb sizes, encoding *tetA(P)* and *tetB(P)*, *cnaC* and toxin gene *cpb2*, with the 50-kb plasmids missing ~20 genes due to transposases encoded by *CPR_0630* on both flanks. Plasmid VIf, encoded by strain VIf, was not detected in strains from other lineages apart from a lineage V strain IQ074 (obtained from infant N10; Fig. 4c), indicating potential plasmid transmissible events between *C. perfringens* strains, because the NICU stay of N10 overlapped with infants carrying VIf strains (Fig. 4d).

The pCP13 plasmids (~45–60 kb) in our dataset harboured *cpb2*, *becAB* and *edpA* toxin genes^29^. Adhesin *cnaB* was detected in all extracted pCP13 plasmids. Importantly, sublineage IIIb strains carried pCP13 plasmids (54,591 bp) which encoded *becAB* binary toxin genes, previously associated with human gastroenteritis, with further analysis confirming identical plasmids from three individual infants (Fig. 4e and Extended Data Fig. 1b).

Another pCP13 plasmid (~62 kb) was detected in nine *C. perfringens* identical isolates (Fig. 4f), obtained from four individuals, that nested in three distinct sublineages in lineage V, highlighting the common transferable nature of conjugative plasmids among *C. perfringens* strains (Fig. 4g). Both plasmids pCW13 and pCP13 were less prevalent in *C. perfringens* strains in lineage V (19%); in contrast plasmid carriage in other lineages ranged from 50% to 86% (Fig. 4h). Plasmid carriage in CPA-NEC strains was 80% versus non-CPA-NEC strains at 44% (Fig. 4i).

### Toxicity assays links toxin gene *pfoA* to epithelial damage

**Fig. 5:**
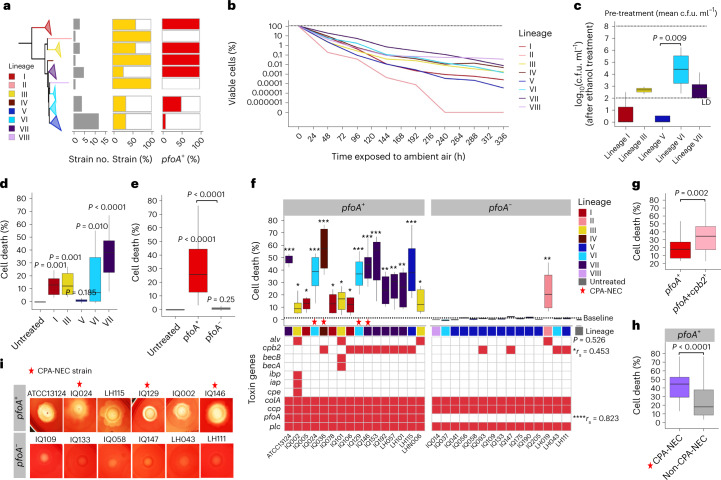
Phenotypic virulence assessment of 30 infant-associated *C. perfringens* strains suggests *pfoA* as a key virulence gene in gut epithelial cell death. **a**, Schematic of strain selection (*n* = 31). **b**, Viability of *C. perfringens* strains (*n* = 31) exposed to ambient air over 14 d (data stratified by lineage). A lineage II strain was undetectable after 10 d. Data are the mean. **c**, Sporulation capacity of *C. perfringens* strains (*n* = 28) assessed using ethanol pre-treatment (4 h) to eliminate vegetative cells before culturing, with *n* = 3 biologically independent samples for each strain. The statistical significance was pairwise compared across the groups. LD, limit of detection. **d**, Toxicity of sterile-filtered supernatants (*n* = 31) determined by 2-h co-culturing with colonic cell line Caco-2 (*n* = 6 in two independent experiments). Cell death comparison was between lineages. The significance was compared with the untreated group. **e**, Cell toxicity of *C. perfringens* strains compared between *pfoA*^+^ (*n* = 15) and *pfoA*^−^ groups (*n* = 15). The *pfoA*^+^ strains encode *pfoA* toxin gene; the *pfoA*^−^ strains do not encode *pfoA*. The significance was compared with the untreated group, and pairwise between *pfoA*^+^ and *pfoA*^−^ groups as indicated. **f**, Cell death percentage of individual strains aligned with toxin gene profiles. The presence of both toxin genes *pfoA* (*r*_s_ = 0.823) and *cpb2* (*r*_s_ = 0.453) was positively correlated with the increase in Caco-2 cell death (*P* < 0.05). A correlation analysis was performed using the point-biserial-correlation test (Spearman’s rank correlation). The significance was compared with the untreated group: ^***^P < 0.001, ^**^P < 0.01, ^*^P < 0.05. **g**, Cell death comparison between *pfoA*^+^ strains (*n* = *7*) and *pfoA*^+^*cpb*2^+^ strains (*n* = 9) showing potential synergistic effects of *cpb2* toxin gene alongside *pfoA*. **h**, Cell death comparison between CPA-NEC *pfoA*^+^ strains (*n* = 4) and non-CPA-NEC *pfoA*^+^ strains (*n* = 26). **i**, Images of *C. perfringens* colonies (10 μl of confluent cultures per spot) on CBA after 24 h of anaerobic incubation. All representative *pfoA*^+^ strains (*n* = 6) exhibit complete haemolysis on CBA whereas *pfoA*^−^ strains (*n* = 6) display partial haemolysis. In **c**–**h**, the box in the boxplot represents 50% of the central data, in between the lower and upper quartiles, with a central line representing the median, whereas the whiskers show the most extreme data points. All statistical analyses were performed using the Kruskal–Wallis test and post-hoc Dunn’s test of multiple comparisons, with *P* values adjusted using the Benjamini–Hochberg method, unless otherwise stated. Significance was tested against the untreated group, otherwise significance brackets were used in pairwise comparison. Source data

From the genomic data, it was apparent that *C. perfringens* strains from lineage V encoded significantly fewer virulence factors, most clearly evidenced by the absence of the *pfoA* toxin gene. We selected 30 infant-associated *C. perfringens* strains from all lineages, including *pfoA*^+^ and *pfoA*^−^ strains for in vitro tests (Fig. 5a).

**Extended Data Fig. 2:**
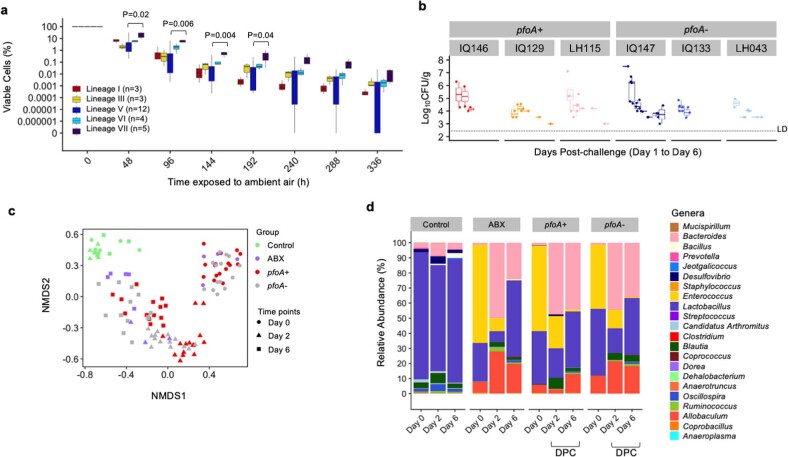
Strain-level in vitro and in vivo assessment of *C. perfringens* virulence potentials. **a**, Oxygen tolerance test. Comparison of viability of *C. perfringens* strains (*n* = *31*) from lineages I, III, V, VI and VII exposed to ambient air (aerobic) over 14 days in 3 biologically independent samples for each strain. Cell viability in percentage was shown from 48 h to 192 h between lineage VII and lineage V with statistical difference. The box in boxplot represents 50% of the central data, in between lower and upper quartile, with a central line representing the median while whiskers show the most extreme data points (also in **b**). **b**, Intestinal colonisation of *C. perfringens* monitored over 7 days post-challenge. LD, limit of detection. **c**, NMDS plot of murine genus-level gut microbiome profiles on day 0, day 2 and day 6. **d**, Comparison of genus-level murine gut microbiome on day 0, day 2 and day 6. A total of 4 groups were compared, Control (*n* = *5*), ABX (*n* = *5*), *pfoA*+ (n = 15) and *pfoA*- (*n* = *15*). DPC: days post-challenge. (All statistical analyses were performed with Kruskal-Wallis test and post-hoc Dunn’s test of multiple comparisons with P values adjusted by Benjamini-Hochberg method. The box in boxplots represents 50% of the central data, in between first and third quartile, with an inner line representing the median). Source data

Survivability/transmissibility assays indicated that lineage VII strains appeared to be most oxygen tolerant (Fig. 5b), surviving significantly better than strains from lineage V from 48 h to 192 h (Extended Data Fig. 2a), although *C. perfringens* strains from lineage VI produced significantly more spores than strains nested in lineage V (Fig. 5c).

*C. perfringens*-associated cellular toxicity assays showed supernatants of strains in lineages I, III, VI and VII inducing significant cell death in epithelial monolayers, whereas lineage V strains did not (Fig. 5d). Grouping by presence of the *pfoA* pore-forming toxin gene indicated a clear cytoxicity signal, whereas cell death induced by *pfoA*^−^ strains was no different statistically from untreated controls (Fig. 5e). Cellular toxicity phenotypes correlated with the presence of *pfoA*, and to a lesser extent toxin gene *cpb2* (Fig. 5f), with a potential synergistic effect observed in strains encoding both toxins (Fig. 5g). It is interesting that *pfoA*^+^ and CPA-NEC strains also exhibited significantly higher cell toxicity versus non-CPA-NEC strains (Fig. 5h). All *pfoA*^+^ strains also exhibited complete haemolysis on blood agar, whereas *pfoA*^−^ strains displayed partial haemolysis (Fig. 5i). One *pfoA*^−^ strain (LH019) induced higher cell death (Fig. 5f), probably due to the presence of alveolysin (*alv*) which shares ~86% homology (nucleotide identity) with *pfoA*^30^.

### *C. perfringens* strains induce murine colonic histopathology

**Fig. 6:**
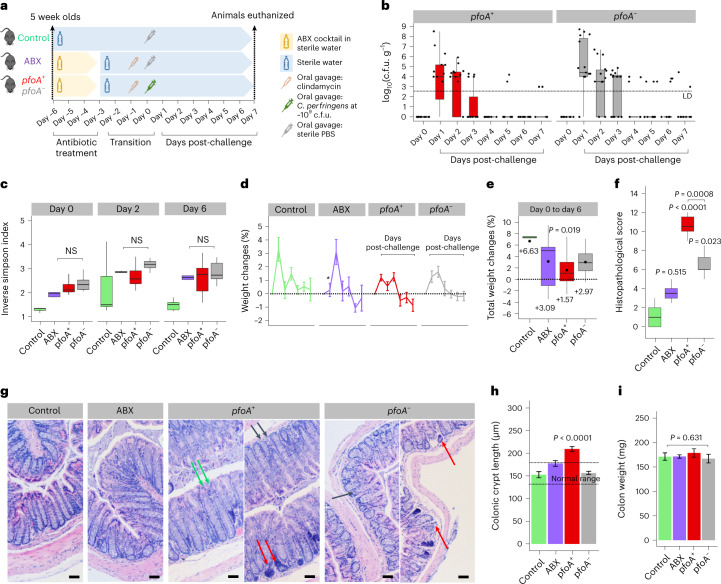
*C. perfringens* strains induce histopathological changes in murine colons. This experiment was based on eight groups of mice (*n* = 5 for each group) including control (untreated) and antibiotic groups, and six experimental groups (three groups of *pfoA*^+^ strains and three groups of *pfoA*^−^ strains). **a**, Schematic of in vivo infection model experimental design. PBS, phosphate-buffered saline. **b**, Intestinal colonization of *C. perfringens* monitored over a 7-d post-challenge *pfoA*^+^ (*n* = 15) versus *pfoA*^−^ (*n* = 15). **c**, Inverse Simpson’s index: gut microbiome diversity of mice on days 0, 2 and 6, respectively, showing no significant genus-level differences when comparing groups *pfoA*^+^ (*n* = *1*5) and *pfoA*^−^ (*n* = 15) to antibiotics *(n* = 5). The statistical analysis was performed with ANOVA and Tukey’s test (post-hoc pairwise). NS, non-significant (*P* > 0.05). **d**, Daily weight changes (%) of mice over experimental period across groups. Data are mean ± s.e.m. ^*^P = 0.034. **e**, Total average weight changes from day 0 to day 6. The *pfoA*^+^ group (*n* = 15) has a significantly lower mean weight change compared with the control group (*P* < 0.05). The statistical analysis was performed with ANOVA and Tukey’s test (post-hoc pairwise). **f**, Histopathological scores for intestinal inflammation of murine distal colons (day 7 post-challenge), comparison between groups. Scoring was based on two H&E-stained images per distal colon. Increments in score value denote increasing severity. **g**, Representative H&E-stained murine distal colonic sections of control, antibiotics and experimental groups (*pfoA*^+^ and *pfoA*^−^), respectively, showing epithelial changes. The green arrow shows erosion and crypt hyperplasia, the black arrow loss of goblet cells and the red arrow immune cell infiltration. Scale bars, 50 µm. **h**, Distal colonic crypt length comparison. Measurement was performed based on 20 crypts per representative image. Data are mean ± s.e.m. Normal range for distal colonic crypt length is 133–175 µm. **i**, Colon weight (whole-colon) comparison across all groups. Data are mean ± s.e.m. In **b**, **c**, **e** and **f**, the box in the boxplot (where applicable) represents 50% of the central data, in between the lower and upper quartiles, with a central line representing the median, whereas the whiskers show the most extreme data points. All statistical analyses were performed using the Kruskal–Wallis test and a post-hoc Dunn’s test of multiple comparisons and *P* values adjusted using the Benjamini–Hochberg method, unless otherwise stated. The significance was tested against the control group; otherwise significance brackets were used in pairwise comparison. Source data

We developed an oral-challenge murine model to explore pathology related to *pfoA* using six *C. perfringens* (*pfoA*^+^/*pfoA*^−^) strains (Fig. 6a). Colonization rates indicated that most strains were completely cleared by day 4 (Fig. 6b and Extended Data Fig. 2b), although microbiota genus-level diversity profiles between *pfoA*+ and *pfoA*^−^ groups did not differ statistically (Fig. 6c and Extended Data Fig. 2c,d). Differences in daily weight changes between groups were not observed (Fig. 6d); however, overall weight change indicated that the *pfoA*^+^ groups had significantly less weight gain versus controls (Fig. 6e).

**Extended Data Fig. 3:**
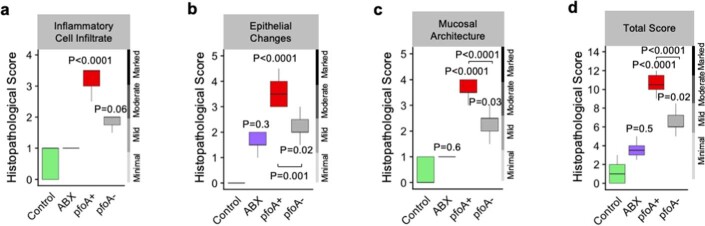
Histopathological analysis on murine distal colonic sections. **a-d**, Histopathological scores for intestinal inflammation of murine distal colons comparison between groups in three main general criteria (features), with indicative severity scale (minimal, mild, moderate and marked). There were 8 groups of mice (*n* = *5* each group) including control groups (Control and ABX) and 6 experimental groups (3 groups of *pfoA*+ and 3 groups of *pfoA*-). The box in boxplot represents 50% of the central data, in between lower and upper quartile, with a central line representing the median while whiskers show the most extreme data points. All statistical analyses were performed with Kruskal-Wallis test and post-hoc Dunn’s test of multiple comparisons with P values adjusted by Benjamini-Hochberg method, unless otherwise stated. Significance was tested against untreated group, otherwise significance brackets will be used in pairwise comparison. **a**, Inflammatory infiltrate: immune cell infiltration (score value: 0–4). **b**, Epithelial changes: epithelial hyperplasia and goblet cell loss (score value: 0–5). **c**, Mucosal architecture: villous blunting (score value: 0–5). **d**, Total score (additive score value: 0–14). Increments in score value denotes increasing severity. Scoring was based on two H&E-stained images per distal colon. Source data

**Extended Data Fig. 4:**
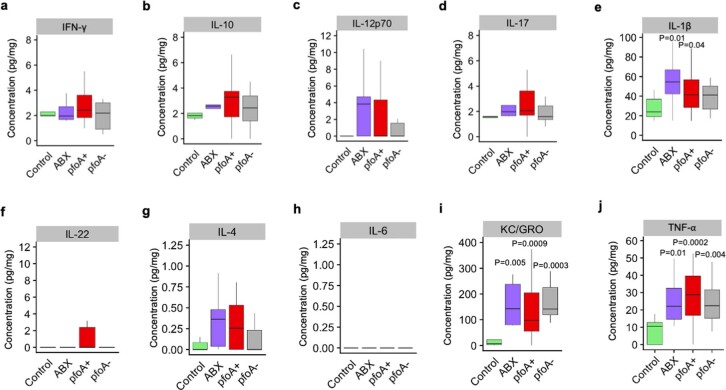
Evaluation of pro-inflammatory cytokine profiles in proximal murine colons. **a-j**, Concentration (pg/mg) of cytokines IFN-γ (**a**), IL-10 (**b**), IL-12p70 (**c**), IL-17 (**d**), IL-1β (**e**), IL-22 (**f**), IL-4 (**g**), IL-6 (**h**), KC/GRO (**i**) and TNF-α (**j**) respectively, in murine proximal colonic samples, stratified into groups: Control (*n* = *5*), ABX (*n* = *5*), *pfoA*+ (*n* = *15*), *pfoA-* (*n* = *15*). All individual samples were measured in technical duplicates (*n* = *2*). The box in boxplot represents 50% of the central data, in between lower and upper quartile, with a central line representing the median while whiskers show the most extreme data points. All statistical analyses were performed with Kruskal-Wallis test and post-hoc Dunn’s test of multiple comparisons with P values adjusted by Benjamini-Hochberg method, unless otherwise stated. Significance was tested against untreated group, otherwise significance brackets will be used in pairwise comparison. Source data

Both *C. perfringens*-infected groups (*pfoA*^+^ and *pfoA*^−^) exhibited significantly higher histopathological scores for intestinal inflammation compared with control groups, with colonic sections from *pfoA*^+^ groups displaying more severe damage/pathology versus *pfoA*^−^ groups (Fig. 6f). Distal colonic sections of both infected groups demonstrated similar pathological features, including marked goblet cell loss and inflammatory cell infiltrate (Fig. 6g and Extended Data Fig. 3), with *pfoA*^+^ groups having significantly longer crypt lengths versus *pfoA*^−^ and control groups (Fig. 6h), although not when comparing colon weights (Fig. 6i). Immune profiling of colonic tissue showed significantly increased levels of tumour necrosis factor (TNF)-α, KC/GRO and interleukin (IL)-1β in the *pfoA*^+^ group versus controls (Extended Data Fig. 4) and, although not significant, elevated levels of IL-22 and IL-6 were observed.

## Discussion

Genomic analysis of preterm infant-associated *C. perfringens* strains suggests distinct lineages that comprise potentially hypovirulent strains versus typical virulent strains linked with possible NICU circulation and carriage of virulence-carrying mobile elements, including CPA-NEC strains. Certain genotype-linked phenotypical traits, for example, *pfoA*^+^-associated cytotoxicity, appear to correlate with increased intestinal cell pathology, which may be associated with infection/disease outcomes.

Currently, public genome databases are biased towards diseased-associated isolates, with only a limited number of *C. perfringens* genomes obtained from healthy individuals (~5%)^31,32^. *C. perfringens* is also considered as a normal gut microbiota member^33,34^ and we observed a potentially hypovirulent or ‘commensal-like’, human-derived monophyletic lineage (V). Strains encoded significantly fewer virulence factors and plasmids and were thus distinct from more typically virulent *C. perfringens*, including CPA-NEC strains. Indeed, potentially hypovirulent lineage V strains may not encode the necessary virulence traits required to cause overt disease such as severe intestinal colitis or CPA-NEC.

A genomic signature apparently enriched in typical, virulent, infant-associated *C. perfringens* isolates (including CPA-NEC strains) was the presence of the gene *pfoA* that transcribes the pore-forming haemolytic toxin PFO. We consistently observed a direct correlation between the presence of *pfoA* and significantly enhanced cell toxicity in intestinal cell lines and complete haemolysis, when compared with *pfoA*^−^ strains regardless of lineage (Fig. 5e,i). PFO (θ-toxin), a typical cholesterol-dependent cytolysin, is known as a pore-forming toxin^35^, which has been heavily associated with the pathogenesis of myonecrosis (gas gangrene), haemorrhagic enteritis in calves and more recently septicaemia (intravascular haemolysis) in humans, including neonates^36–40^. PFO can act synergistically with α-toxin in the pathology of gas gangrene, which is a disease that shares a high degree of symptom similarity with CPA-NEC in preterm infants, and has also been called ‘intestinal gas gangrene’^19,41,42^. The haemolysis rates of *C. perfringens* septicaemia clinical isolates were shown to strongly correlate with PFO expression alone, regardless of α-toxin production (whereas a *pfoA*-deficient strain did not induce haemolysis), with proinflammatory cytokine production (TNF-α, interleukin IL-5 and IL-6) significantly stronger than α-toxin^40^. Nevertheless, the mechanistic role of PFO in disease development is not completely defined, although previous studies have indicated additional modulation of host responses^36,43–45^. Indeed, our in vivo studies suggest a high degree of colonic immune cell infiltration and a higher proinflammatory cytokine milieu (and associated pathology) during *pfoA*^+^ *C. perfringens* infection. Although previous studies have indicated that the *pfoA* toxin gene is universally encoded in *C. perfringens*, aside from gastroenteritis-associated toxinotype F strains (nested in lineage VIII in Fig. 1a), we did not observe this in lineage V strains^8,9,46,47^. This prior assumption may be due to skewing in public databases for disease-associated isolates, so our data highlight the important role of this toxin in disease involvement, including CPA-NEC.

A concern with typical virulent *pfoA*^+^ *C. perfringens* strains is nosocomial transmission in at-risk populations, which is a huge issue in related *Clostridioides difficile*^48,49^. We observed a circulating strain VIf, which was detected in six individuals during their NICU stay, and seemingly circulated between two sister hospitals that regularly exchange patients. Transmission of *C. perfringens* has also been demonstrated in similar confined settings such as within elderly care homes (more vulnerable communities), where non-foodborne outbreaks of toxigenic *C. perfringens* take place frequently^8,50^. Notably, the index case of strain VIf was detected in an individual who had been treated with broad-spectrum antibiotics, suggesting that creation of a more ‘favourable’ niche may have allowed *C. perfringens* to flourish in the microbiota-depleted gut. This strain (IQ129) also nested within lineage VI linking to a stronger spore-forming capacity (Fig. 5c), a trait that may provide protection against antibiotics, as demonstrated by its detection 8 d after termination of antibiotic treatment. Although six infants harboured strain VIf, only N20 developed CPA-NEC and ultimately succumbed to the infection. This preterm infant had been previously diagnosed with congenital heart disease which may have caused reduced blood flow and oxygen supply to the gut, potentially altering the gut microbiota and barrier permeability that may favour the growth of certain virulent *C. perfringens* strains^51–53^. Further studies exploring wider microbiome dynamics longitudinally in preterm infant cohorts, complemented by in vitro studies (for example, model colon systems), are required to tease apart potential mechanisms.

The widespread dissemination of certain strains may be partly attributed to several traits. Typical *pfoA*^+^ *C. perfringens* strains (including those associated with CPA-NEC) appeared to be more oxygen tolerant and have enhanced spore-forming/germination ability, which may facilitate spread between infants and NICUs. This may include resilience against standard disinfectant practices, thus enabling persistence in even adverse ‘sterile’ hospital environments, and subsequent germination once spores enter the infant gut^54,55^. These traits pose a significant challenge for infection control measures, especially those wards where extremely low-birth-weight, preterm infants reside who have a heightened risk of developing CPA-NEC^56^. The presence of circulating strains also indicates that routine genomic surveillance of *C. perfringens* may be helpful in NICUs to monitor and prevent circulation of virulent strains.

Certain plasmids (for example, pCW3 and pCP13) have long been recognized as key mobile genetic elements for transfer of virulence genes in *C. perfringens*^27,28^. We observed nine isolates from three distinctive sublineages in lineage V carrying identical pCP13 conjugative plasmids, suggesting frequent conjugative plasmid transfer^28^. Alongside virulence factors, certain colonization factors are encoded on these plasmids such as adhesin genes *cnaB* and *cnaC*^27^, with collagen adhesin critical in pathogenesis by conferring additional cell-binding ability in host strains^57^. Notably, adhesin gene *cnaC*, also required for conjugative transfer, has previously been correlated with virulent poultry-NE *C. perfringens* strains, whereas a pCP13 plasmid that encodes *cnaB* and *becAB* was recently linked to acute gastroenteritis outbreaks^6,27,30,58^. Carriage of these plasmids was found to be more common in all lineages except hypovirulent lineage V, suggesting that plasmid-encoded virulence traits represent key genomic signatures linked to pathophysiology. This potential plasmid circulation links with recent findings that phylogenetically distinct type-F *C. perfringens* isolates were found to harbour identical CPE-encoding plasmids in a single gastroenteritis outbreak^8^. However, as our results are based on short-read sequence data (albeit at very high sequencing coverage), further studies using long-read-based sequencing with plasmid reconstruction/characterization are required to probe these findings in detail.

We identified *pfoA* as the major toxin gene positively associated with intestinal cell injury, whereas *pfoA*^+^ CPA-NEC strains also correlated with significantly higher cell death compared with *pfoA*^+^ non-CPA-NEC strains. Notably, overt infection and subsequent production of virulence factors in many cases will be prevented by gut microbiota colonization resistance mechanisms. However, the preterm infant gut microbiota is known to be significantly disrupted compared with full-term infants (in which NEC is extremely rare), which may allow overgrowth of *C. perfringens*^1^. The use of probiotics has been proposed as a prophylactic strategy to beneficially modulate the preterm gut microbiota and reduce incidence of NEC, and previous studies have indicated that supplementation with the early life microbiota genus *Bifidobacterium* spp. correlated with a reduction in *Clostridium* spp. and associated NEC^59,60^.

We added an antibiotic regimen (five-antibiotic cocktail + clindamycin) to deplete the murine microbiota, and more closely mimic the clinical scenario in preterm neonates, who are routinely exposed to empirical antibiotic therapy^59^. Distinctive disease symptoms were detected: *pfoA*^+^ strains induced moderate colonic inflammation versus milder colonic inflammation observed in the *pfoA*^−^ groups, suggesting that PFO may act as a major virulence factor linked to intestinal pathology. The pathological changes observed in distal colonic sections also correlated with diarrhoea, which is similar to *Citrobacter rodentium* infection, a model for human enteropathogenic *Escherichia coli*^61,62^. It also appeared that immune-mediated pathology plays a role during *pfoA*^+^ *C. perfringens* infection, as evidenced by mucosal erosions, goblet cell reduction, immune cell infiltration and increased levels of the proinflammatory cytokine TNF-α, when compared with *pfoA*^−^-infected animals. This implicates the importance of the potential immune-driven aspects of *C. perfringens* intestinal infection, which warrants further investigation. Although *pfoA*^+^ CPA-NEC strains were used in this infection model, we did not observe severe CPA-NEC symptoms including complete intestinal necrosis and abdominal distension. This is probably due to differences between mice and humans and factors such as diet. The standard chow diet may not favour this protein-hungry pathogen, the use of adolescent mice (rather than neonatal mice) may lead to more efficient *C. perfringens* clearance through immune-mediated pathways, and murine rather than preterm gut microbiota may have impacted infection kinetics. Further optimization studies, including the use of ‘humanized’ mouse models, may allow development of a more clinically relevant CPA-NEC model^63^.

There are certain limitations associated with the present study. First, only five CPA-NEC strains from four CPA-NEC patients from two sister hospitals were included. Given the very rare occurrence of CPA-NEC (subset of NEC cases), we had access to a limited number of patients (including issues/restrictions related to the severe acute respiratory syndrome coronavirus 2 (SARS-CoV-2) pandemic), and we were unable to reconstruct any additional *C. perfringens* metagenome-assembled genomes (MAGs) from previously published preterm infant microbiome studies, which was largely due to lack of sequencing depth^64^. Within our dataset/cohort we had ~6% NEC incidence. A rate of between 5% and 15% NEC is often reported, but its incidence varies widely between NICUs, with NEC cases often reported in ‘outbreaks’ that may link to emergence of a specific virulent strain in the hospital environment, a hypothesis supported by our SNP analysis^60,65^. This is particularly important, because previous terminology for the disease ‘intestinal gas gangrene’ signals the more severe and fulminant condition of CPA-NEC, compared with classic NEC, often leading to surgery within 24 h and associated higher mortality rates (78%)^19,66^. Larger (and longer) surveillance studies that incorporate numerous NICUs across a wider geographical region would be required to capture a larger number of samples from CPA-NEC-diagnosed infants. We also fully recognize NEC as a multifactorial intestinal disease that has been associated with several bacteria including *C. perfringens* and *Klebsiella* and *Enterococcus* spp.^21,22,67^. Indeed, two preterm neonates had previously been diagnosed with *Klebsiella*-associated NEC in the previous cohort study^22^ and, although they each harboured *C. perfringens* strains, these isolates exhibited a ‘commensal’ genomic signature (within hypovirulent lineage V and did not encode *pfoA*). We used Caco-2 cells to examine whether the secreted toxins of *C. perfringens* caused epithelial damage. We appreciate the usual caveats of using an immortal cell line and over-extrapolation of results; however, our further strain testing in vivo does strengthen our findings in terms of cell death and links to pathology. Future work is required to pinpoint the mechanistic role of the virulence factors identified, such as *pfoA*, potentially in a more clinically relevant, preterm infant enteroid model^68^ and/or using neonatal mice, although ethical and regulatory considerations limit these types of studies.

Overall, we have identified a potentially hypovirulent lineage of *C. perfringens*, characterized by lack of the *pfoA* toxin gene, colonization factors and survival-related capacities, in contrast to the typical *pfoA*^+^ virulent lineages of *C. perfringens* (including CPA-NEC strains), which linked to in vitro and in vivo pathological traits. Potential dissemination of virulent *C. perfringens* strains was observed between and within preterm infants and hospitals (including a case of CPA-NEC), highlighting the potential value of routine surveillance and enhanced infection control measures.

## Methods

### Clinical samples and bacterial isolation work

We conducted a retrospective genomic analysis on *C. perfringens* isolates obtained from faecal samples of 70 neonatal patients admitted to NICUs at hospitals A, B, C, D and E, respectively, in the UK between February 2011 and March 2016 (Supplementary Table 2). All cohort data and sequenced microbiota profiles can be accessed in the original clinical study publications; most samples were sequenced via 16S ribosomal RNA or metagenomic sequencing as per the original studies/publications in which the colonization dynamics had already been reported and discussed^22,59,69^. Dates of hospital admission and transfers were extracted electronically. Faecal samples collected were stored at −80 °C in a freezer before the experimental process to isolate *C. perfringens* using the ethanol-shock method (50% ethanol in Robertson’s cooked meat medium), followed by plating on fastidious anaerobic agar supplemented with defibrinated sheep blood and 0.1% sodium taurocholate; alternatively, faecal samples were plated directly on tryptose–sulfite–cycloserine egg yolk agar (TSC-EYA) before 37 °C anaerobic incubation for 18–24 h (ref. ^70^). Multiple or single distinct colonies on the plates were purified and maintained as pure isolates in autoclaved brain–heart infusion (BHI) broth with 30% glycerol for cryopreservation at −80 °C. Subjects without *C. perfringens* were defined as *C. perfringens* negative by culturing. We predefined CPA-NEC in the present study as definite NEC diagnosis (Bell stage II/III) with overabundant *C. perfringens* before NEC onset (Bell’s staging classification system: Bell stage II/III is considered to be definite NEC whereas Bell stage I covers non-specific signs) as described in Sim et al.^22^. This was a 2-year microbiota study that involved a cohort of >350 infants, with 4 CPA-NEC cases who showed statistically enriched *C. perfringens* before NEC onset in longitudinal samples (by comparing with samples from aged-matched non-NEC preterm infants), although CPA-NEC strains were the isolates collected at the final time points before disease onset.

### Genomic DNA extraction and WGS

Pure isolates were cultured anaerobically overnight at 37 °C in BHI broth (~10–15 h) for genomic DNA extraction using either the phenol–chloroform extraction method or FastDNA SPIN Kit for soil according to the manufacturer’s instructions (hospital E samples only; MP Biomedicals). For the phenol–chloroform extraction method^71^, briefly, overnight 10 ml of pure cultures in BHI was harvested, followed by resuspension of bacterial pellets in 2 ml of 25% sucrose in 10 mM Tris and 1 mM EDTA, pH 8.0. Cells were then enzymatically lysed using 50 μl of lysozyme, 100 mg ml^−1^ (Roche). Next, 100 μl of Proteinase K, 20 mg ml^−1^ (Roche), 30 μl of RNase A, 10 mg ml^−1^ (Roche), 400 μl of 0.5 M EDTA, pH 8.0 and 250 μl of 10% Sarkosyl NL30 (Thermo Fisher Scientific) were added accordingly into the lysed suspension. This was then followed by 1 h of ice incubation and a 50 °C water bath overnight. On the second day, three rounds of phenol–chloroform–isoamyl alcohol (Merck) extraction were performed using 15 ml of gel-lock tubes (QIAGEN). Chloroform–isoamyl alcohol (Merck) extraction was then carried out followed by ethanol precipitation and 70% ethanol wash (once). DNA pellets (usually visible) were finally resuspended in 200–300 μl of 10 mM Tris, pH 8.0 and stored at −20 °C until further analysis. WGS of each isolate sample was performed on Illumina HiSeq 2500 to generate 101 125-bp paired-end reads^72^. Isolate samples from hospital E were sequenced on Illumina NextSeq 500 to generate 150-bp paired-end reads.

### De novo genome assemblies and metagenome-assembled genomes

Raw sequence reads (FASTQ) generated from sequencers were quality filtered (-q 20) with fastp v.0.20.0 before de novo genome assembly using SPAdes v.3.14.1 (ref. ^73^) at default parameters. Contigs of <500 bp were discarded in each isolate genome assembly before subsequent analyses. Genome assembly statistics were generated via sequence-stats v.1.0 (ref. ^74^) (Supplementary Table 3). All infant-associated genome assemblies were subjected to contamination check via CheckM v.1.1.3 (ref. ^75^) and contaminated (contamination >5%) and/or incomplete (completeness <90%) genome assemblies were excluded from further analysis (*n* = 2). A total of 117 human-gut-associated, metagenome-assembled genomes and 17 isolate genomes of *C. perfringens* was retrieved from the Unified Human Gastrointestinal Genome v.2.0 (UHGG)^76^ collection (genome completeness >80% and contamination <5%).

### Phylogenetic analyses

A total of 673 *C. perfringens* genome assemblies was investigated, including 171 public isolate genomes retrieved from GenBank, 272 infant-associated draft genomes newly generated in the present study, 96 food-poisoning-linked isolate genomes (from the UK) published previously and 117 high-quality metagenome-assembled genomes, alongside 17 isolate genomes from UHGG (human-gut-associated *C. perfringens* genomes)^8,76^. All genomes investigated in the present study passed contamination checks via CheckM v.1.1.3 (ref. ^75^) (completeness >80% and contamination <5%) and taxonomically assigned as *C. perfringens* by gtdb-tk v.1.5.1 (ref. ^77^) (average nucleotide identity (ANI) > 95% against type strain).

These high-quality *C. perfringens* genome assemblies (*n* = 673) were subsequently dereplicated at strain level (ANI > 99.9%) using dRep v.3.2.2 (ref. ^78^) before reconstruction of a core-gene alignment. A core-gene alignment (comprising 858 single-copy core genes) of 310 *C. perfringens* genomes based on Prokka v.1.14.6 (ref. ^79^) annotation of coding sequences (general feature format) was next generated using Panaroo v.1.2.8 (ref. ^80^) with options --merge_paralogs, --clean-mode strict and --aligner mafft^81^. In a similar manner, infant-associated, core-gene alignment (1,995 single-copy core genes) was generated using dereplicated strain-level genomes (*n* = 80) via Panaroo v.1.2.8. SNPs were next extracted from core-gene alignments using snp-sites v.2.3.3 (ref. ^82^) and further dereplicated at core-gene level via in-house script, resulting in 302 representative genomes in the global phylogenetic tree (Fig. 1) and 80 representative genomes in the infant-associated phylogenetic tree (Fig. 2; with two reference genomes, type strains ATCC13124 and NCTC8679). Maximum-likelihood trees were constructed based on the extracted and dereplicated core-gene SNP alignment using IQ-TREE v.2.0.5 (ref. ^83^) with ultra-fast bootstrap replicates -B 1000 and automatic evolutionary model selection option -m TEST (best fit model for both trees was determined as GTR + F + ASC + G4).

Phylogenetic lineages were assigned using R library RhierBAPS v.1.1.3 (ref. ^84^). Tree topology was checked and validated using mash-distance trees generated by Mashtree v.1.2.0 (ref. ^85^). Tree annotation was performed via iTOL v.6.5.8 (ref. ^86^).

### Toxinotype assignment and virulence profiling

Toxinotypes A–G were assigned to each *C. perfringens* sample via database TOXIper v.1.1 (ref. ^87^). Virulence-related genes and colonization factor sequence search were performed via ABRicate v.1.0.1 with options --minid=90 and *--*mincov=80 based on in-house sequence databases (Supplementary Table 5). ResFinder v.4.0 database was used via ABRicate for profiling antimicrobial resistance genes^88^.

### SNP analysis

SNPs were extracted from core-gene alignment with snp-sites v.2.3.3 (ref. ^82^) and snp-dists v.0.7 (ref. ^89^) was utilized for computing pairwise SNP distances. Transmission analysis was limited to hospital-associated strains (from 70 individuals) and within closest genetic distance, identified by phylogenetic topologies and pairwise SNP distances within 8,506 core-gene SNPs in 858 single-copy core-gene alignment. Probable transmission dynamics were predicted based on clinical metadata of the patients and the time of the individuals’ stay within the hospitals. SNP distance was further explored to determine the exact genetic distance to infer transmission.

### Plasmid investigation

Plasmid analysis was limited to infant-associated *C. perfringens* genomes. Conjugative plasmids, both pCW3 and pCP13 families (known to encode multiple virulence factors), were extracted based on the identification of the *tcp* (pCW3 plasmids^27^; *n* = 12) and *pcp* (pCP13 (ref. ^28^) plasmids; *n* = 20) genes in the conjugative systems via ABRicate v.1.0.1 (ref. ^90^). Briefly, *tcp* and *pcp* loci and plasmid replication protein gene *rep* were comprehensively searched on 274 genomes; only single contigs within genome assemblies comprising more than five conjugative genes (*rep* is compulsory) were extracted and assumed as functional conjugative plasmids for further analyses. Predicted genes were mapped to reference plasmids LH112 (the largest plasmid size in both families) to allow genome architecture comparison of plasmid contents across both pCW3 and pCP13 plasmid families. Virulence factors were identified via ABRicate v.1.0.1 with in-house databases as described in the previous section. Plasmid sequences were aligned with MAFFT v.7.305b, SNP distance was compared via snp-dists v.0.7 (ref. ^81^). Easyfig v.2.2.2 (ref. ^91^) was utilized for visualization of plasmid sequence comparison.

### Maintenance of cell line Caco-2

Frozen stocks were resuscitated/resuspended in pre-warmed (37 °C) Dulbecco’s Modified Eagle Medium (DMEM; Thermo Fisher Scientific), supplemented with 20% fetal bovine serum (FBS; Thermo Fisher Scientific). Cells were immediately transferred to T75 sterile culture flasks (Corning) and incubated at 37 °C in a 5% CO_2_ incubator for 24 h. Spent medium was removed after 48–72 h, replaced with 20–25 ml of fresh warm medium as described and incubated until confluency was reached at ~5–8 d. Caco-2 cell line passages 53–54 were used for the present study, cells were split at ratios 1:5–10 for each passage. Cells were counted on a haemocytometer (Neubauer Chamber).

### In vitro phenotypic characterization

#### Cell toxicity assay

The Caco-2 cell line was maintained in liquid nitrogen at Quadram Institute Bioscience (American Type Culture Collection (ATCC), catalogue no. HTB-37). Cells (passages 53–54) were seeded at 20,000 cells per well in tissue-treated 96-well plates (Corning Costar). After 6–7 d of 5% CO_2_ incubation at 37 °C, a confluent monolayer was formed (manually confirmed using microscopy) and subjected to further experiments. Briefly, phenol-red DMEM was removed, cells were washed twice gently and replaced with phenol-red-free DMEM supplemented with 1% FBS. Diluted sterile-filtered (0.22-μm filter) bacterial supernatants (selected *C. perfringens* strains were cultured from 1:100 confluent overnight cultures for 10 h before collection of supernatants; all strains reached a density of ~10^9^ colony-forming units (c.f.u.) ml^−1^ at the end of 10 h) were added neat into each well at 12.5% total volume followed by a 2-h 5% CO_2_ incubation at 37 °C. Cell death measurement (fluorescence signal of lactate dehydrogenase released from cells with a damaged cell membrane) was performed and estimated using CytoTox-ONE Homogeneous Membrane Integrity Assay according to the manufacturer’s instructions (Promega) using a black 96-well plate to reduce background readouts (Corning).

### Haemolysis assay

Columbian blood agar (CBA; 5% sheep blood; Oxoid) was used to identify the haemolytic trait of *C. perfringens* isolates linked to pore-forming toxins produced. Briefly, *C. perfringens* was streaked on the blood agar and incubated anaerobically overnight (<20 h). Isolated colonies with clear transparent halos formed in and around the colonies indicate complete haemolysis. Assay outcomes were observed manually.

### Oxygen tolerance assay

Pure cultures of 31 *C. perfringens* strains (including 30 infant-associated isolates and type strain ATCC13124) were grown anaerobically to confluency for 24 h in BHI. Subsequently, cultures were spotted in a dilution series on to BHI agar supplemented with 0.1% sodium taurocholate (potent germinant for spores). Spotted Petri dishes were dried and incubated under ambient (aerobic) conditions at room temperature (22 °C) for specified time periods before being returned to an anaerobic chamber for determination of the colony-forming unit count (maximum 14 d). All colony-forming units were counted after 15–20 h of anaerobic incubation at 37 °C. Cultures (spotted plates) that were not exposed to ambient conditions acted as controls for each strain. A viable percentage was shown after comparing with control cultures.

### Sporulation assay

Cultures of 31 *C. perfringens* strains (including 30 infant-associated isolates and type strain ATCC13124) were induced to sporulate using the modified Duncan-strong medium (proteose peptone 16 g l^−1^, yeast extract 4 g l^−1^, cysteine hydrochloride 0.5 g l^−1^, disodium phosphate 10 g l^−1^ and raffinose 4 g l^−1^, with a final pH of 7.8) for 24-h anaerobic incubation using 1:100 overnight confluent cultures^92^. Cultures were pre-treated with sterile-filtered 70% ethanol for 4 h to eliminate vegetative cells and spot plated on BHI agar supplemented with 0.1% taurocholate (potent germinant) for spores. Agar plates were incubated anaerobically for 24 h before counting the colony-forming units.

### In vivo studies

#### Ethics and licence

All animal experiments and related protocols described were performed under the Animals (Scientific Procedures) Act 1986 (ASPA) under project licence no. PP8873233 and approved by the Home Office and University of East Anglia (UEA) FMH Research Ethics Committee. Animals are monitored and assessed frequently during studies for physical condition and behaviour. Mice determined to have suffered from distress would be euthanized via the ASPA schedule 1 protocol (CO_2_ and cervical dislocation). Trained and qualified animal technicians carried out animal husbandry at UEA Disease Modelling Unit (DMU).

### Animals, housing and animal study design

Specific pathogen-free C57BL/6 female mice (*n* = 40; 3-week-old *Mus musculus*), obtained from Charles River (France) were used in the animal study. Animals were transported to and housed in the UEA DMU acclimatization barn under specific pathogen-free conditions for 2 weeks before moving to the infection suite. During the infection study, mice were housed with autoclaved bedding (and cage), food (stock pellets) and water with a 12 h light cycle (12 h:12 h light:darkness). Cages were changed in a laminar flow cabinet. The rooms were maintained at 21 ± 2 °C and 55 ± 10% humidity. Air change rates are 12–15 per h.

Mice were caged into eight groups (five mice in each group), which include control group, ABX group (only treated with antibiotics) and six experimental groups (*pfoA*^+^ versus *pfoA*^−^). Group *pfoA*^+^ (*n* = 3) only was challenged with *C. perfringens* strains IQ146, IQ129 and LH115, which encode a *pfoA* toxin gene, whereas group *pfoA*^−^ (*n* = *3*) was given only *C. perfringens* strains that do not encode the *pfoA* toxin gene, IQ147, IQ133 and LH043, respectively.

Experimental groups (*n* = 6) and the ABX group were treated with a five-antibiotic cocktail comprising kanamycin (0.4 mg ml^−1^), gentamicin (0.035 mg ml^−1^), colistin (850 U ml^−1^), metronidazole (0.215 mg ml^−1^) and vancomycin (0.045 mg ml^−1^) in autoclaved drinking water for 3 d administered freely (Fig. 6a). Drinking water was then switched to sterile water and, 48 h later, mice in the experimental group and the ABX group were orally gavaged with 150 mg kg^−1^ of clindamycin. After 24 h, mice were gavaged with ~10^9^ c.f.u. of *C. perfringens* in 100 μl and all mice were closely monitored for signs of disease symptoms, including significant weight loss (>20%). All animals were euthanized and the organs harvested at day 7 (Fig. 6a).

### Bacterial strains and growth

Bacterial stocks were recovered on BHI agar for purity checks each time and subsequently cultured in BHI broth overnight to reach confluency (~10^9^ c.f.u. ml^−1^, approximately 14–17 h). Bacterial pellets were washed and resuspended in sterile phosphate-buffered saline before feeding the mice via oral gavaging using a 20G plastic sterile feeding tube.

### Faecal sample collection and colony-forming unit enumeration

Faecal samples were collected in sterilized tubes daily and stored at −80 °C until further analysis. Serially diluted faecal mixtures were plated on fresh TSC agar and the colony-forming units enumerated after <24 h anaerobic incubation. Pitch-black colonies were counted as *C. perfringens* colonies.

### Tissue processing and H&E staining

Distal colons were fixed in 10% neutral buffered formalin (~4% formaldehyde; Merck) for <24 h and followed by 70% ethanol. Tissues were subsequently processed using an automated Leica Tissue Processor ASP-300-S and embedded in paraffin manually. Next, sectioning of tissues was performed using a Leica microtome (5-μm-thick sections) and left overnight for samples to air dry before staining and further analysis. Haematoxylin and eosin (H&E) staining was then performed using Leica Autostainer for structural imaging of intestinal samples.

### Microscopy imaging

Bright-field microscopy was performed using Olympus BX60 with a microscope camera Jenoptik C10 and ProgRes CapturePro software v.2.10 (Supplementary Figs. 1–8).

### Histopathological scoring

All distal colonic section images (two to three distal colonic images per animal) were examined and graded single-blinded by L.J.H. The histological severity of intestinal inflammation was graded using a histopathological scoring system (0–14; denoting increasing severity) based on three general pathological features: inflammatory cell infiltrate (score value: 0–4), epithelial changes that include epithelial hyperplasia and goblet cell loss (score value: 0–5) and mucosal architecture—villous blunting (score value: 0–5) as described by Erben et al.^93^. The overall additive score (0–14) was the sum of each component score value. An overall pathological score of 1–4 suggests minimal colitis, 5–8 mild colitis, 9–11 moderate colitis and >12 marked/severe colitis.

### Morphometry

Crypt length of distal colons was examined digitally via ImageJ2 v.2.3.0 based on 20 crypts per representative image of distal colonic microscopy slides.

### Cytokine analysis

Proximal murine colons were homogenized in lysis buffer (150 mmol l^−1^ of NaCl, 20 mmol l^−1^ of Tris, 1 mmol l^−1^ of EDTA, 1 mmol l^−1^ of EGTA and 1% Triton X-100 at pH 7.5) using a FastPrep-24 bead-beating grinder (MP Biomedicals) at speed 4.0 for 40 s followed by speed 6.0 for 40 s. Samples were then centrifuged for 12 min at 4 °C. Supernatants were transferred to clean tubes and further quantified (protein concentration) before cytokine analysis.

Protein concentration was quantified by BCA (bicinchoninic acid) assay (BioRad) and cytokines were analysed using a customized Mesoscale Discovery U-Plex assay (MSD) according to the manufacturer’s instructions. Cytokine concentrations were normalized to total protein input and samples were run in duplicates. The plate was read using an MSD QuickPlex SQ 120 imager and data analysed on the MSD Discovery Workbench software.

### Gut microbiome analysis

Genomic DNA of murine faecal samples was extracted, sequenced and analysed following the protocol described previously^6^. Briefly, DNA was extracted from samples using FastDNA Spin Kit for Soil (MP Biomedicals) following the manufacturer’s instruction, while extending the bead-beating (in FastPrep-24 bead-beating grinder) step to 80 s at speed 6.0 (ref. ^59^). Next, genomic DNA extracts were subjected to the 16S rRNA gene V1 + V2 region library preparation before sequencing on an Illumina NovaSeq sequencing platform at 2 × 250 bp (paired-end sequencing)^59^. Sequencing raw reads (FASTQ) were first merged using PEAR v.0.9.6 (ref. ^94^), then underwent quality and chimera filtering via QIIME v.1.9.1 (ref. ^95^), subsequently with operational taxonomic unit (OTU) assignment using a SILVA_132 database^96^. BIOM output of OTU tables was read using MEGAN6 (ref. ^97^).

### Data visualization

Phylogenetic trees (mid-point rooted and unrooted) were graphed using iTOL v.6.5.8 (ref. ^86^). Various statistical graphs including pie charts, line charts, dot plots, bar plots and box plots were drawn in R v.4.1.2 (ref. ^98^), using R libraries tidyverse v.1.3.1 (ref. ^99^), ggplot2 v.3.3.5 (ref. ^100^) and ggpubr v.0.4.0 (ref. ^101^). R library vegan v.2.6.2 (ref. ^102^) was used to construct non-metric multidimensional scaling (NMDS) plot for microbiome data and estimation of the inverse Simpson’s index.

### Sample size, randomization and blinding

No statistical methods were used to predetermine sample size for experiments. Sample size for the in vivo study was selected following the 3Rs principles—reduction, replacement and refinement—ensuring sufficient sample size for meaningful statistical analysis.

Randomization was not attempted during the in vivo study to avoid cross-contamination because treatments were performed according to the individual cages; however, animals were initially allocated to each cage randomly. For in vitro experiments, randomization is not relevant due to the way bacterial isolates were selected with an aim of formally characterizing *pfoA*^+^ isolates (*n* = 15) and *pfoA*^−^ isolates (*n* = 15).

Single blinding was used in the in vivo colon tissue histopathological scoring analysis to prevent bias. For in vivo sample collection and analysis, the investigators were blinded to group allocation. For other in vitro experiments, data collection and analysis were not performed blind to the conditions of the experiments.

### Data exclusion

Two *C. perfringens* isolate genomes (sequenced in the present study) were excluded from genome analysis due to detected sequence contamination by CheckM v.1.1.3 (>10%). No animals or data points were excluded from analyses.

### Statistics and reproducibility

Statistical tests were performed via R base packages stats v.4.1.2 (ref. ^98^), including Fisher’s exact test (two sided), Kruskal–Wallis test, Wilcoxon’s test, analysis of variance (ANOVA), post-hoc Tukey’s test and point-biserial correlation test. The Shapiro–Wilk normality test was used to formally test for data normality where appropriate. R library rstatix v.0.7.0 (ref. ^103^) was used for post-hoc Dunn’s test of multiple comparisons (two sided), whereas rcompanion v.2.4.16 (ref. ^104^) was utilized to conduct pairwise post-hoc tests (Fisher’s exact test).

For both oxygen tolerance and sporulation assays, experiments were performed on three biologically independent replicates per sample (bacterial strain; *n* = 3). In cell toxicity assay, cell death measurements were performed in two independent experiments, each in three biological replicates per sample (sterile supernatants of bacterial strains; *n* = 6). The animal study was conducted once with five C57BL/6 mice each group or cage (*n* = 40). For murine distal colonic samples, two to three subsections of each processed distal colon (cross-sections) were photographed using a digital bright-field microscope and used for further phenotypic examinations.

### Ethics statement

Faecal collection from Norfolk and Norwich University Hospital (NNUH) and Rosie Hospital (BAMBI study) was approved by the Faculty of Medical and Health Sciences Ethics Committee at the UEA and followed protocols laid out by the UEA Biorepository (licence no. 11208). Faecal collection from Imperial Healthcare NICUs (NeoM and NeoM2 studies) was approved by West London Research Ethics Committee (REC) under the REC approval reference no. 10/H0711/39. In all cases, doctors and nurses recruited infants after parents had given written consent. Ethical approval for the SERVIS study was approved by the North East and N Tyneside committee 10/H0908/39, and signed parental consent was obtained from every parent. We have anonymized identifiers of patients, hospitals and associated clinical data.

### Reporting summary

Further information on research design is available in the Nature Portfolio Reporting Summary linked to this article.

## Supplementary information

Supplementary Information Supplementary Figs. 1–8.

Reporting Summary

Supplementary Tables Supplementary Table 1 Clinical statistics: a summary of sample screening, *C. perfringens* isolates and incidence (colonization data) in this multi-center collaborative study. Supplementary Table 2 Clinical metadata. Supplementary Table 3 Genome assembly statistics. Supplementary Table 4 Metadata for in vivo metagenomics study. Supplementary Table 5 GenBank accessions for virulence genes. Supplementary Table 6 Links to databases used in the present study.

## Data Availability

Genome assemblies of 171 *C. perfringens* isolates were retrieved from the National Center for Biotechnology Information’s (NCBI) GenBank (downloaded on 2 April 2020), while 96 *C. perfringens* food-poisoning-associated, isolated genomes were downloaded (May 2022) from the European Nucleotide Archive under accession no. PRJEB25764 (Supplementary Table 3). Another 117 *C. perfringens* metagenome-assembled genomes and 17 *C. perfringens* isolated genomes were retrieved from the UHGG v.2.0 collection (May 2022; Supplementary Table 3). Sequencing raw reads and draft genome assemblies for 272 *C. perfringens* isolates generated in the present study are publicly available in the NCBI Sequence Read Archive (SRA) and GenBank respectively, under accession no. PRJNA755973 (Supplementary Table 3). The 16S rRNA gene amplicon sequence reads (in vivo microbiome study) are publicly available in SRA under accession no. PRJNA755974 (Supplementary Table 4). Accessible links to sequence databases including ResFinder v.4.0, TOXIper v.1.1, *tcp* loci, *pcp* loci and *C. perfringens*-associated virulence genes (Supplementary Table 5) used in the present study are available in Supplementary Table 6. Anonymized clinical metadata have been made available in Supplementary Table 2. Computationally extracted plasmid sequences, histology images and data used for the figures (Source data files) are listed in the inventory (‘Inventory_of_supplementary_info.xlsx’) which is openly shared via the GitHub repository: https://github.com/raymondkiu/Infant-Clostridium-perfringens-Paper (ref. ^105^). Source data are provided with this paper.

## Source data

Source Data Fig. 1 Statistical source data.
Source Data Fig. 2 Statistical source data.
Source Data Fig. 3 Statistical source data.
Source Data Fig. 4 Statistical source data.
Source Data Fig. 5 Statistical source data.
Source Data Fig. 6 Statistical source data.
Source Data Extended Data Fig. 1 Statistical source data.
Source Data Extended Data Fig. 2 Statistical source data.
Source Data Extended Data Fig. 3 Statistical source data.
Source Data Extended Data Fig. 4 Statistical source data.

## Code availability

R scripts associated with data visualization and statistical analysis are available and shared via GitHub (https://github.com/raymondkiu/Infant-Clostridium-perfringens-Paper)^105^.
