# Supplementary material for: Particular genomic and virulence traits associated with preterm infant-derived toxigenic Clostridium perfringens strains
Source: Nat Microbiol. 2023 May 25;8(6):1160–75. doi: 10.1038/s41564-023-01385-z (PMC10234813; doi:10.1038/s41564-023-01385-z)
Supplement: Supplementary file 1 — Supplementary Figs. 1–8. [file 41564_2023_1385_MOESM1_ESM.pdf]

nature microbiology

Article https://doi.org/10.1038/s41564-023-01385-z

Particular genomic and virulence traits associated with preterm infant-derived toxigenic *Clostridium perfringens* strains

In the format provided by the authors and unedited

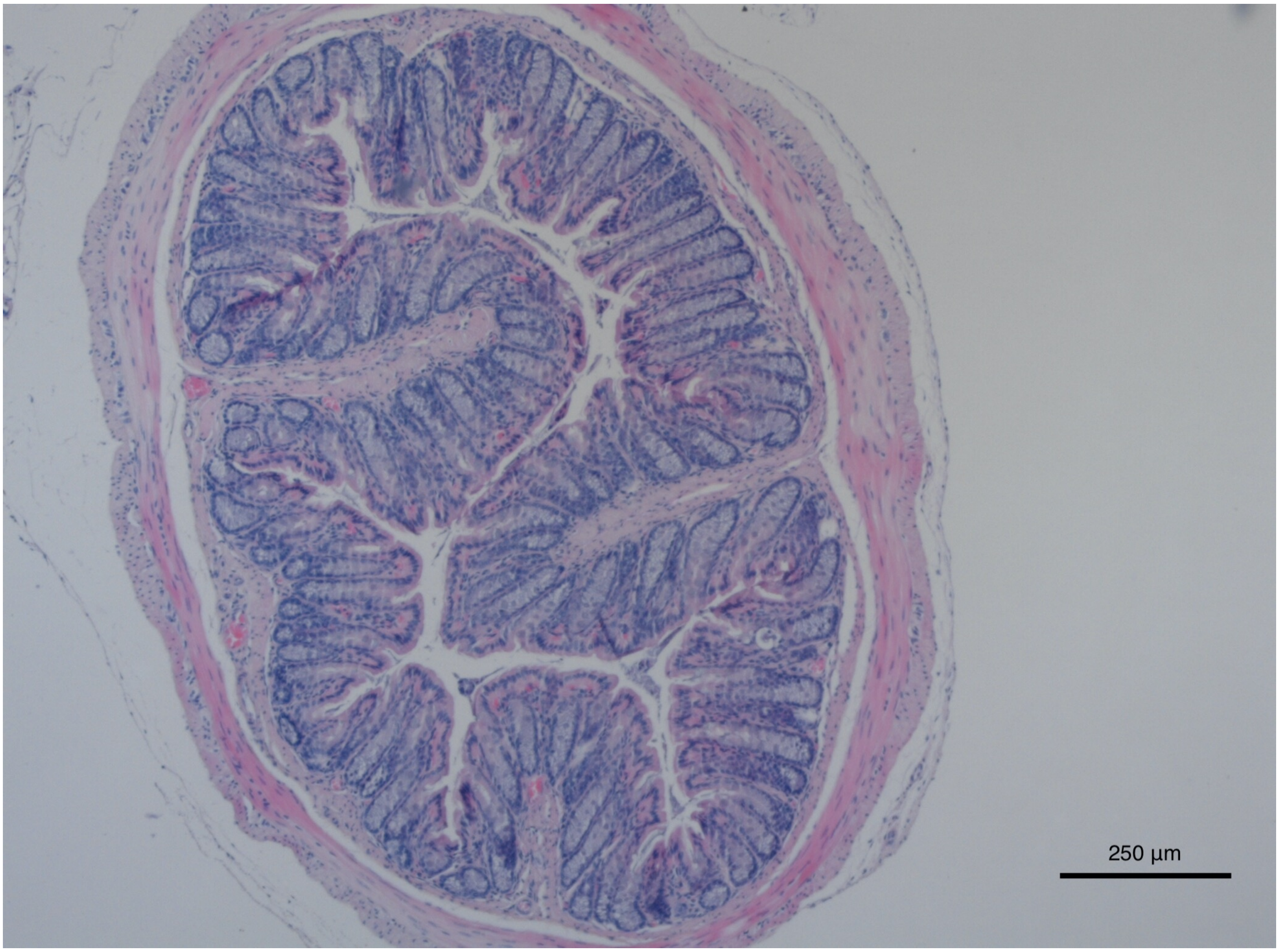

Supplementary Fig. 1. Representative H&E-stained murine distal colonic section of Control group.

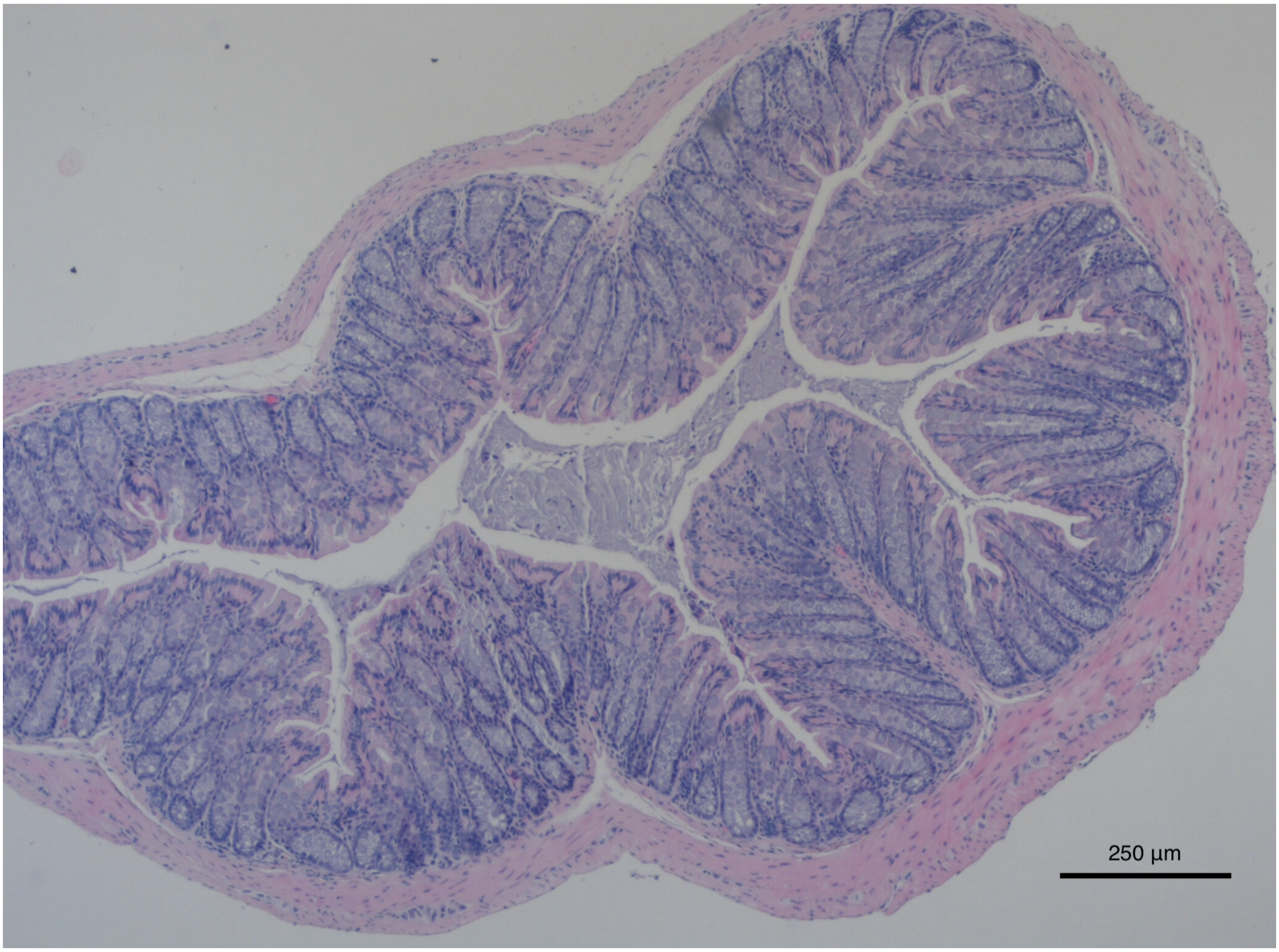

Supplementary Fig. 2. Representative H&E-stained murine distal colonic section of ABX group.

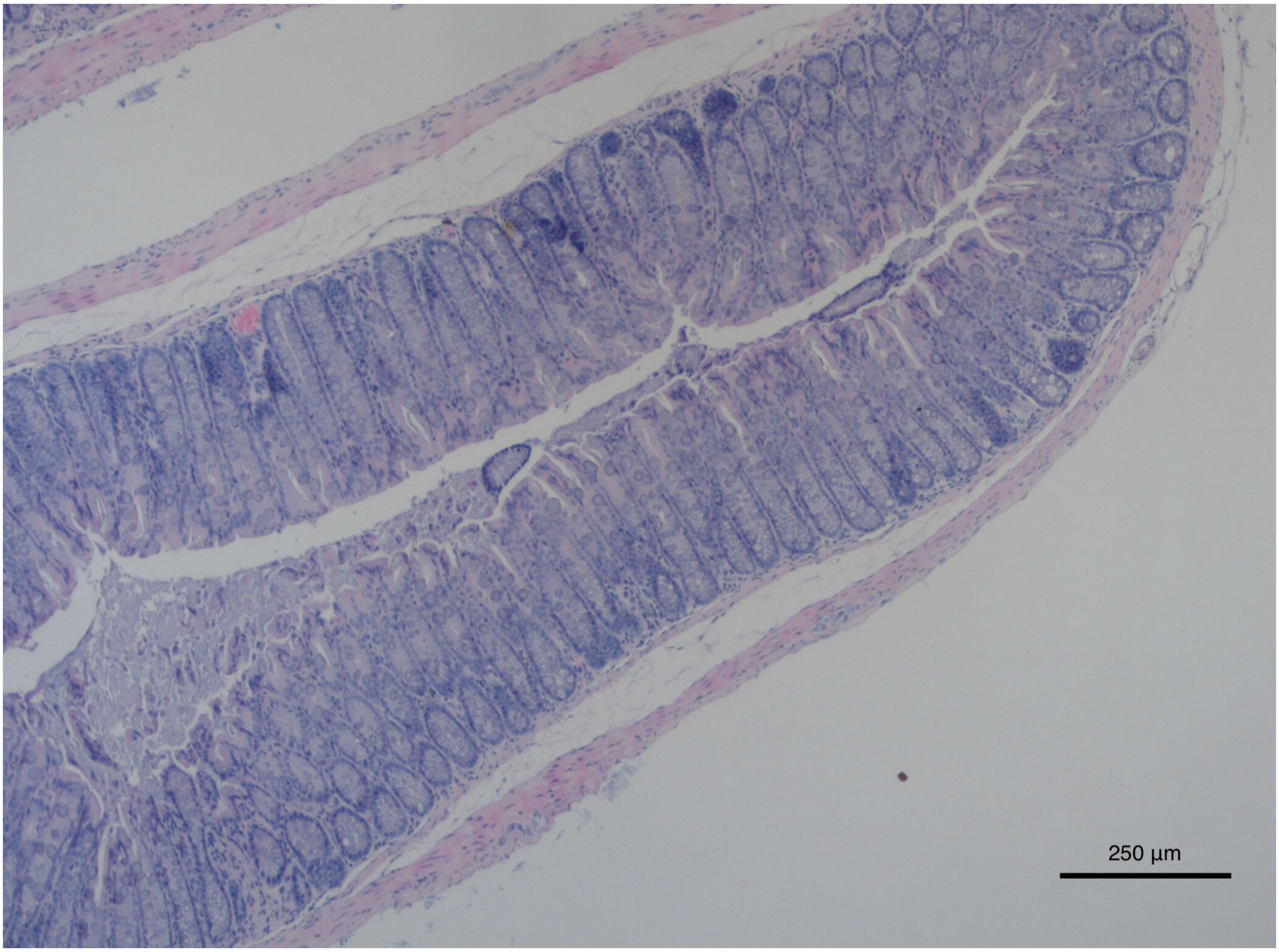

Supplementary Fig. 3. Representative H&E-stained murine distal colonic section of *pfoA*+ strain IQ146.

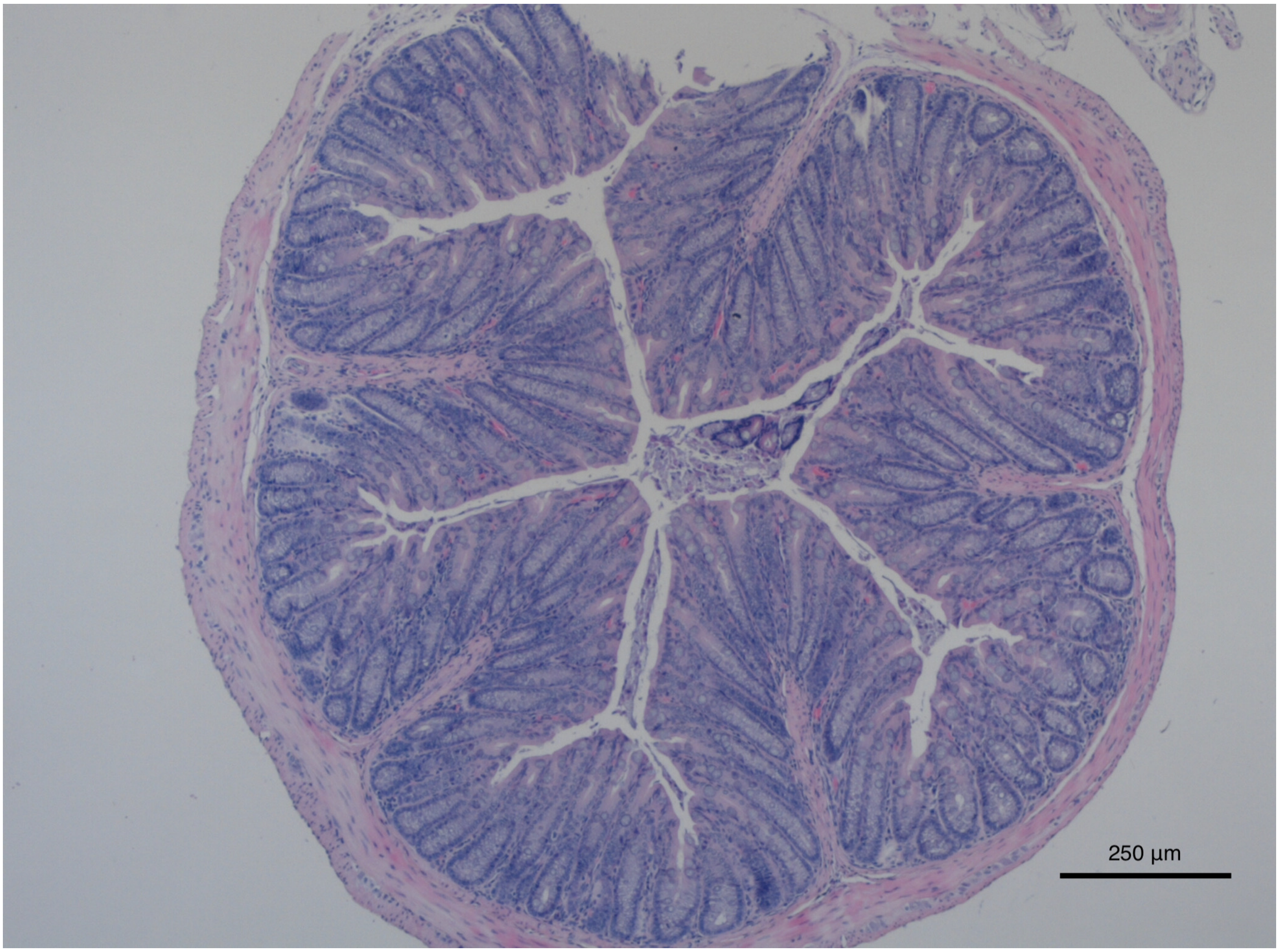

Supplementary Fig. 4. Representative H&E-stained murine distal colonic section of *pfoA*+ strain IQ129.

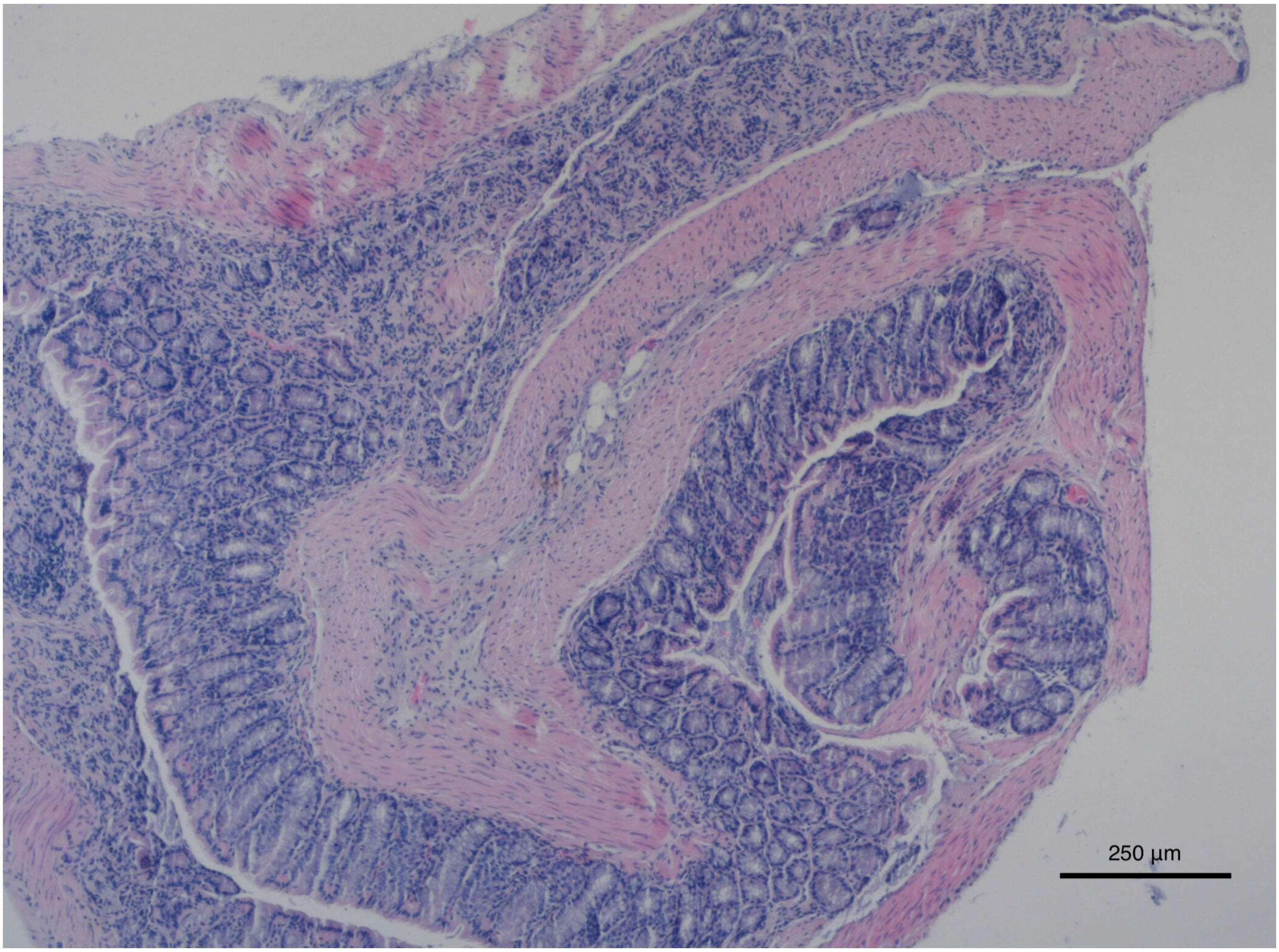

Supplementary Fig. 5. Representative H&E-stained murine distal colonic section of *pfoA*+ strain LH115.

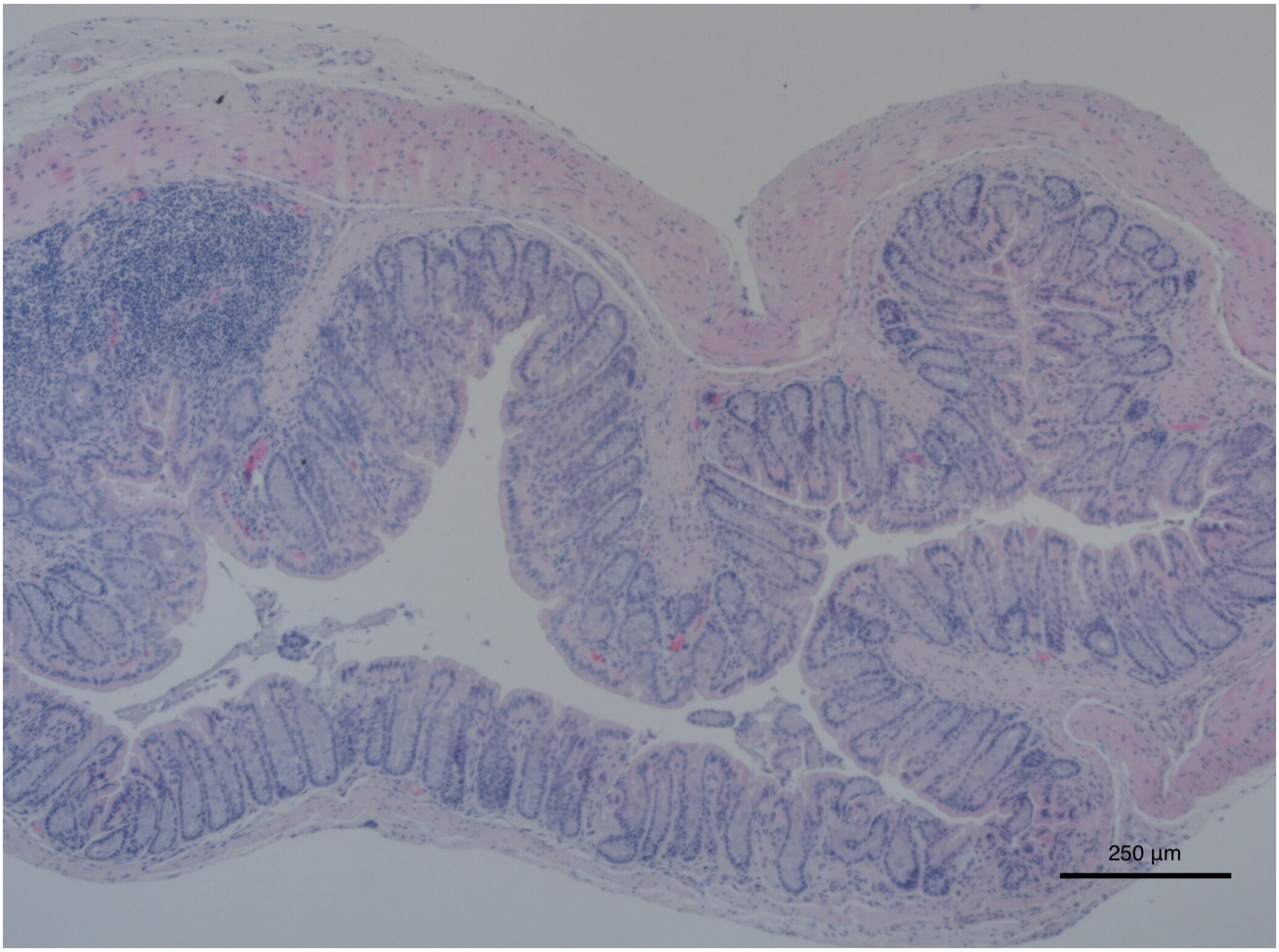

Supplementary Fig. 6. Representative H&E-stained murine distal colonic section of *pfoA-* strain LH043.

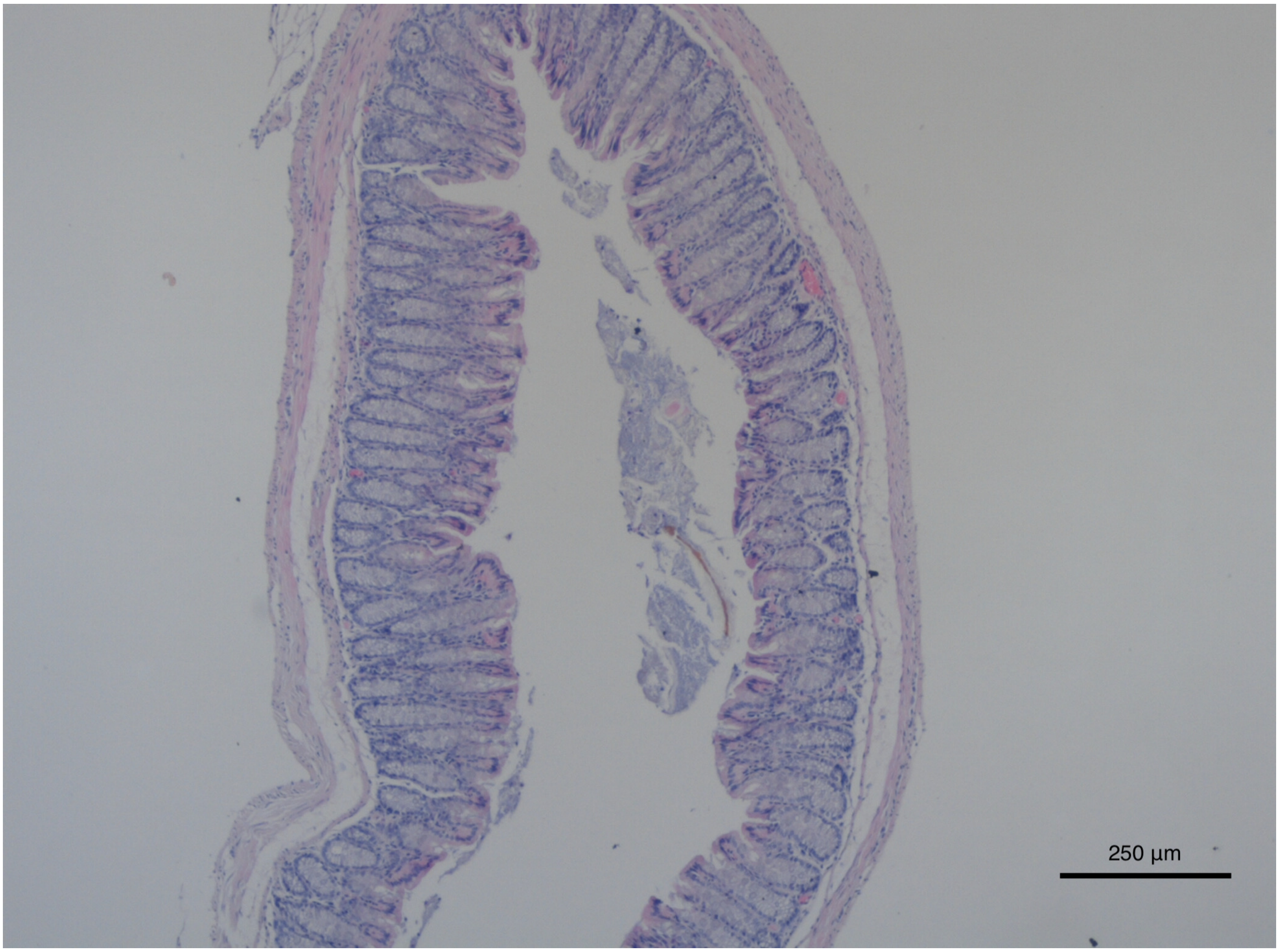

Supplementary Fig. 7. Representative H&E-stained murine distal colonic section of *pfoA-* strain IQ147.

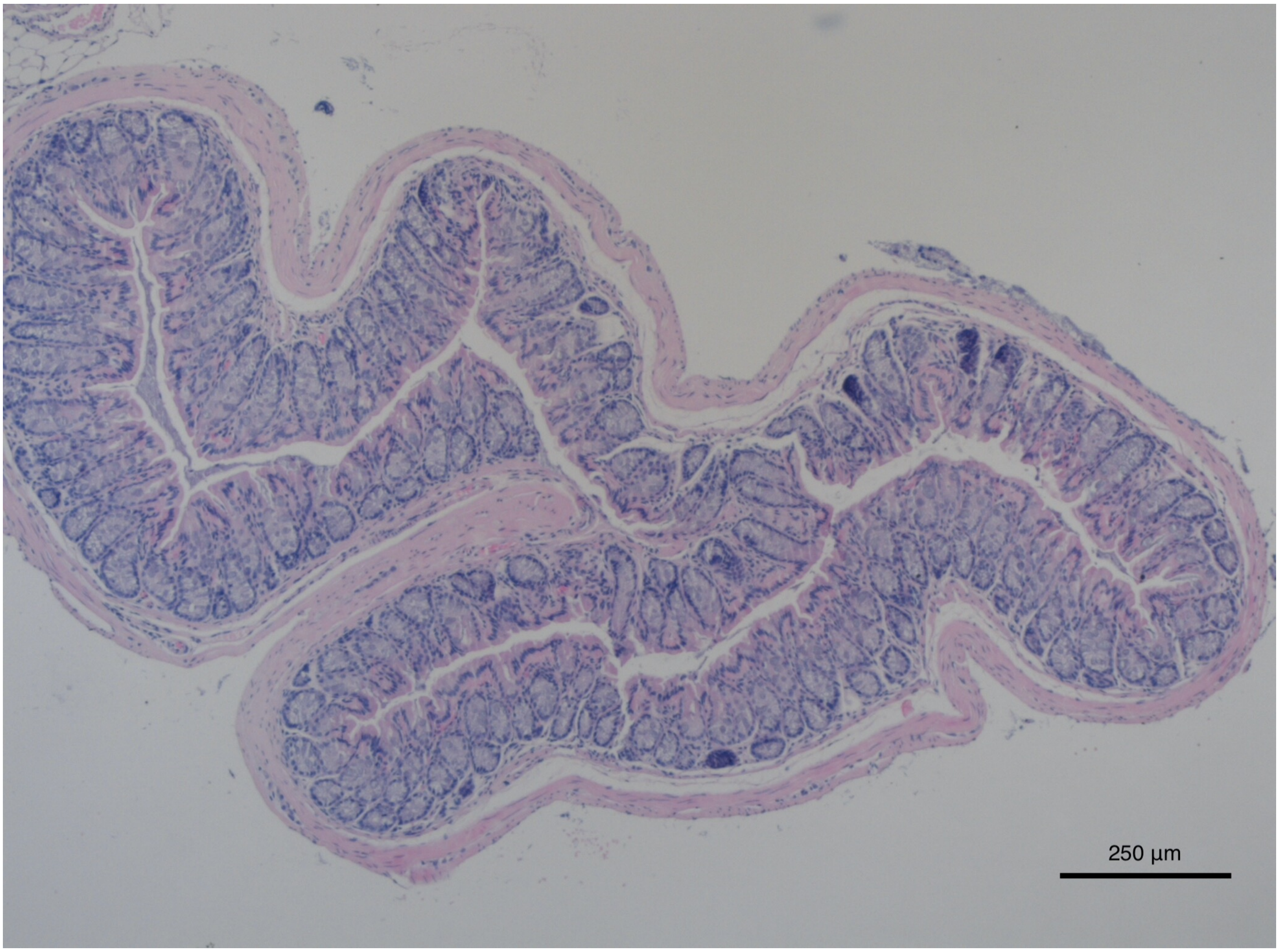

Supplementary Fig. 8. Representative H&E-stained murine distal colonic section of *pfoA-* strain IQ133.
