# Supplementary material for: Particular genomic and virulence traits associated with preterm infant-derived toxigenic Clostridium perfringens strains
Source: Nat Microbiol. 2023 May 25;8(6):1160–75. doi: 10.1038/s41564-023-01385-z (PMC10234813; doi:10.1038/s41564-023-01385-z)
Supplement: Supplementary file 2 — Reporting Summary [file 41564_2023_1385_MOESM2_ESM.pdf]

nature portfolio

Corresponding author(s): Prof. Lindsay J Hall

Last updated by author(s): Mar 17, 2023

Reporting Summary

Nature Portfolio wishes to improve the reproducibility of the work that we publish. This form provides structure for consistency and transparency in reporting. For further information on Nature Portfolio policies, see our Editorial Policies and the Editorial Policy Checklist.

Statistics

For all statistical analyses, confirm that the following items are present in the figure legend, table legend, main text, or Methods section.

n/a	Confirmed	
☐	☒	The exact sample size (*n*) for each experimental group/condition, given as a discrete number and unit of measurement
☐	☒	A statement on whether measurements were taken from distinct samples or whether the same sample was measured repeatedly
☐	☒	The statistical test(s) used AND whether they are one- or two-sided<br>*Only common tests should be described solely by name; describe more complex techniques in the Methods section.*
☒	☐	A description of all covariates tested
☐	☒	A description of any assumptions or corrections, such as tests of normality and adjustment for multiple comparisons
☐	☒	A full description of the statistical parameters including central tendency (e.g. means) or other basic estimates (e.g. regression coefficient) AND variation (e.g. standard deviation) or associated estimates of uncertainty (e.g. confidence intervals)
☐	☒	For null hypothesis testing, the test statistic (e.g. *F*, *t*, *r*) with confidence intervals, effect sizes, degrees of freedom and *P* value noted<br>*Give P values as exact values whenever suitable.*
☒	☐	For Bayesian analysis, information on the choice of priors and Markov chain Monte Carlo settings
☒	☐	For hierarchical and complex designs, identification of the appropriate level for tests and full reporting of outcomes
☒	☐	Estimates of effect sizes (e.g. Cohen's *d*, Pearson's *r*), indicating how they were calculated

Our web collection on statistics for biologists contains articles on many of the points above.

Software and code

Policy information about availability of computer code

Data collection	The dataset of 171 Clostridium perfringens public bacterial genome assemblies were downloaded from NCBI GenBank (on 2 April 2020) for comparison with novel isolate sequences (272) generated in this study. 96 C. perfringens isolate genomes were downloaded (May 2022) from ENA accession PRJEB25764. Another 117 C. perfringens metagenome assembled genomes and 17 C. perfringens isolate genomes were retrieved from the Unified Human Gastroinstestinal Genome v2.0 (UHGG) collection. Genomes were all retrieved via web browser, no specific tools were used.
Data analysis	1) fastp v0.20.0 2) SPAdes v3.14.1 3) Sequence-stats v1.0 4) checkm v1.1.3 5) gtdb-tk v1.5.1 6) dRep v3.2.2 7) Prokka v1.14.6 8) Panaroo v1.2.8 9) snp-sites v2.3.3 10) IQ-TREE v.2.0.5 11) RhierBAPS v1.1.3 12) Mashtree v1.2.0 13) iTOL v6.5.8 14)ABRicate v1.0.1 15) snp-dists v0.7 16) MAFFT v7.305b 17) Easyfig v2.2.2 18) ProgRes CapturePro v2.10 19) ImageJ2 v2.3.0 20) PEAR v0.9.6 21) QIIME v1.9.1 22) MEGAN v6 23) R v4.1.2 24) R tidyverse v1.3.1 25) R ggplot2 v3.3.5 26) R ggpubr v0.4.0 27) R vegan v2.6.2 28) R stats v4.1.2 29) R rstatix v0.7.0 30) R rcompanion v2.4.16. R scripts and source data files associated with data visualisation and statistical analysis are available and shared via GitHub (https://github.com/raymondkiu/Infant-Clostridium-perfringens-Paper)

For manuscripts utilizing custom algorithms or software that are central to the research but not yet described in published literature, software must be made available to editors and reviewers. We strongly encourage code deposition in a community repository (e.g. GitHub). See the Nature Portfolio guidelines for submitting code & software for further information.

Data

Policy information about availability of data

All manuscripts must include a data availability statement. This statement should provide the following information, where applicable:

- Accession codes, unique identifiers, or web links for publicly available datasets
- A description of any restrictions on data availability
- For clinical datasets or third party data, please ensure that the statement adheres to our policy

Genome assemblies of 171 C. perfringens isolates were retrieved from NCBI GenBank (downloaded on 2 April 2020), while 96 C. perfringens food-poisoning associated isolate genomes were download (May 2022) from European Nucleotide Archive (ENA) under project accession PRJEB25764 (Supplementary Table 3). Another 117 C. perfringens metagenome assembled genomes and 17 C. perfringens isolate genomes were retrieved from the Unified Human Gastrointestinal Genome v2.0 (UHGG) collection (May 2022; Supplementary Table 3). Sequencing raw reads and draft genome assemblies for 272 C. perfringens isolates generated in this study are publicly available in the NCBI Sequence Read Archive (SRA) and GenBank respectively, under project PRJNA755973 (Supplementary Table 3). 16S rRNA gene amplicon sequence reads (in vivo microbiome study) are publicly available in SRA under project PRJNA755974 (Supplementary Table 4).
Accessible links to sequence databases including ResFinder v4.0, TOXIper v1.1, tcp loci, pcp loci and C. perfringens-associated virulence genes (Supplementary Table 5) used in this study are available in Supplementary Table 6. Anonymised clinical metadata has been made available in Supplementary Table 2. Computationally extracted plasmid sequences, histology images, and data used for the figures (source data files) are listed in the inventory (“Inventory_of_supplementary_info.xlsx”) which is openly shared via GitHub repository: https://github.com/raymondkiu/Infant-Clostridium-perfringens-Paper

Human research participants

Policy information about studies involving human research participants and Sex and Gender in Research.

Reporting on sex and gender	*Use the terms sex (biological attribute) and gender (shaped by social and cultural circumstances) carefully in order to avoid confusing both terms. Indicate if findings apply to only one sex or gender; describe whether sex and gender were considered in study design whether sex and/or gender was determined based on self-reporting or assigned and methods used. Provide in the source data disaggregated sex and gender data where this information has been collected, and consent has been obtained for sharing of individual-level data; provide overall numbers in this Reporting Summary. Please state if this information has not been collected. Report sex- and gender-based analyses where performed, justify reasons for lack of sex- and gender-based analysis.*
Population characteristics	*Describe the covariate-relevant population characteristics of the human research participants (e.g. age, genotypic information, past and current diagnosis and treatment categories). If you filled out the behavioural & social sciences study design questions and have nothing to add here, write "See above."*
Recruitment	*Describe how participants were recruited. Outline any potential self-selection bias or other biases that may be present and how these are likely to impact results.*
Ethics oversight	*Identify the organization(s) that approved the study protocol.*

Note that full information on the approval of the study protocol must also be provided in the manuscript.

Field-specific reporting

Please select the one below that is the best fit for your research. If you are not sure, read the appropriate sections before making your selection.

☒ Life sciences ☐ Behavioural & social sciences ☐ Ecological, evolutionary & environmental sciences

For a reference copy of the document with all sections, see nature.com/documents/nr-reporting-summary-flat.pdf

Life sciences study design

All studies must disclose on these points even when the disclosure is negative.

Sample size	For genomic analysis, Clostridium perfringens strains were isolated from longitudinal stool samples of 70 infants residing in hospital NICU, analysed by Whole Genome Sequencing (n=272). An additional 171 C. perfringens genomes were obtained from NCBI GenBank, 96 C. perfringens isolate genomes were downloaded from ENA accession PRJEB25764, 117 C. perfringens metagenome assembled genomes and 17 C. perfringens isolate genomes were retrieved from the Unified Human Gastroinsteinal Genome v2.0 (UHGG) collection. Sample size for in vivo study was selected following 3 R's principles - Reduction, Replacement and Refinement, ensuring sufficient sample size for meaningful statistical analysis. For in vitro assays, both sporulation and oxygen tolerance assays, experiments were performed in 3 biologically independent replicates per sample. For haemolysis assay, also 3 biological replicates were attempted. Specifically for cell toxicity (Caco2 cell line) assays, two separate experiments were performed with 3 biologically independent replicates each sample (in total 6 biological replicates; bacterial strain supernatants) to achieve biologically/statistically meaningful results.
Data exclusions	2 novel isolate genomes were excluded from genome analysis due to detected sequence contamination by checkm software (>10%).

Replication	Cell toxicity assay was performed twice with 3 biological replicates each sample per experiment. For sporulation, oxygen tolerance and haemolysis assays, 3 biological replicates for each sample in single experiments. All attempts of replication were successful and no experimental data were excluded.
Randomization	Randomisation was not possible in in vivo study to avoid cross-contamination as treatments were performed according to the individual cages. Randomisation is not relevant to all other in vitro experiments due to the way isolates were selected with an aim to characterise pfoA+ isolates (n=15) and pfoA- isolates (n=15).
Blinding	Single-blinding was used in in vivo colon tissue histopathological scoring analysis to prevent bias. For in vivo sample collection and analysis, the investigators were blinded to group allocation. For other in vitro experiments, data collection and analysis were not performed blind to the conditions of the experiments.

Reporting for specific materials, systems and methods

We require information from authors about some types of materials, experimental systems and methods used in many studies. Here, indicate whether each material, system or method listed is relevant to your study. If you are not sure if a list item applies to your research, read the appropriate section before selecting a response.

Materials & experimental systems

n/a	Involved in the study
☒	☐ Antibodies
☐	☒ Eukaryotic cell lines
☒	☐ Palaeontology and archaeology
☐	☒ Animals and other organisms
☒	☐ Clinical data
☒	☐ Dual use research of concern

Methods

n/a	Involved in the study
☒	☐ ChIP-seq
☒	☐ Flow cytometry
☒	☐ MRI-based neuroimaging

Eukaryotic cell lines

Policy information about cell lines and Sex and Gender in Research

Cell line source(s)	Caco-2 cell line maintained in liquid nitrogen at Quadram Institute Bioscience (QIB). Commercial source: https://www.atcc.org/products/htb-37
Authentication	Cell lines were validated using microscopy to determine typical cell morphology and structure.
Mycoplasma contamination	Cell lines were not tested for mycoplasma contamination.
Commonly misidentified lines (See ICLAC register)	No commonly misidentified cell line was used in the study

Animals and other research organisms

Policy information about studies involving animals; ARRIVE guidelines recommended for reporting animal research, and Sex and Gender in Research

Laboratory animals	Mus musculus C57BL/6 mice, female, 3-5 weeks old.
Wild animals	This study did not involve wild animals.
Reporting on sex	Sex was not considered in the study design.
Field-collected samples	The study did not involve samples collected from the field.
Ethics oversight	All animal experiments and related protocols described were performed under the Animals (Scientific Procedures) Act 1986 (ASPA) under project licence no. PP8873233 and approved by Home Office and University of East Anglia (UEA) FMH Research Ethics Committee. Animals are monitored and assessed frequently during studies for physical condition and behavior. Mice determined to have suffered from distress would be euthanised via ASPA Schedule 1 protocol (CO2 and cervical dislocation). Trained and qualified animal technicians carried out animal husbandry at UEA Disease Modelling Unit (DMU).

Note that full information on the approval of the study protocol must also be provided in the manuscript.
